# DLST-dependence dictates metabolic heterogeneity in TCA-cycle usage among triple-negative breast cancer

**DOI:** 10.1038/s42003-021-02805-8

**Published:** 2021-11-16

**Authors:** Ning Shen, Sovannarith Korm, Theodoros Karantanos, Dun Li, Xiaoyu Zhang, Eleni Ritou, Hanfei Xu, Andrew Lam, Justin English, Wei-Xing Zong, Ching-Ti Liu, Orian Shirihai, Hui Feng

**Affiliations:** 1 Department of Pharmacology and Experimental Therapeutics, Boston University School of Medicine, Boston, MA USA; 2 Department of Medicine, Section of Hematology and Medical Oncology, Boston University School of Medicine, Boston, MA USA; 3 Department of Medical Oncology, Johns Hopkins Kimmel Cancer Center, Baltimore, MD USA; 4 Department of Biostatistics, Boston University School of Public Health, Boston, MA USA; 5 Department of Medicine, Department of Molecular and Medical Pharmacology, David Geffen School of Medicine, University of California, Los Angeles, CA USA; 6 Department of Chemical Biology, Ernest Mario School of Pharmacy, Rutgers University, Piscataway, NJ USA

**Keywords:** Cancer metabolism, Breast cancer

## Abstract

Triple-negative breast cancer (TNBC) is traditionally considered a glycolytic tumor with a poor prognosis while lacking targeted therapies. Here we show that high expression of dihydrolipoamide S-succinyltransferase (DLST), a tricarboxylic acid (TCA) cycle enzyme, predicts poor overall and recurrence-free survival among TNBC patients. DLST depletion suppresses growth and induces death in subsets of human TNBC cell lines, which are capable of utilizing glutamine anaplerosis. Metabolomics profiling reveals significant changes in the TCA cycle and reactive oxygen species (ROS) related pathways for sensitive but not resistant TNBC cells. Consequently, DLST depletion in sensitive TNBC cells increases ROS levels while N-acetyl-L-cysteine partially rescues cell growth. Importantly, suppression of the TCA cycle through DLST depletion or CPI-613, a drug currently in clinical trials for treating other cancers, decreases the burden and invasion of these TNBC. Together, our data demonstrate differential TCA-cycle usage in TNBC and provide therapeutic implications for the DLST-dependent subsets.

## Abstract (web summary)

Shen and Korm et al. investigate the role of dihydrolipoamide S-succinyltransferase (DLST), a tricarboxylic acid (TCA) cycle enzyme, in the metabolic heterogeneity of triple negative breast cancer (TNBC) cell lines. They show that either depletion or inhibition of DLST in the dependent cells leads to decreased invasion and metastasis in vitro and in vivo.

## Introduction

Triple-negative breast cancer (TNBC) is an aggressive malignancy associated with high recurrence rates for localized disease and increased risk of visceral metastasis, as well as poor prognosis for advanced disease^1,2^. Due to the absence of hormone receptor overexpression and *HER2* amplification, TNBC is not suited for targeted therapy directed against these receptors, with chemotherapy being the main treatment option, especially for those without *BRCA1/2* mutations^1,3^. Moreover, lacking predictive prognostic biomarkers, it remains difficult for clinicians to select effective therapeutic approaches for this disease^4^. An improved understanding of disease etiology could reveal novel cellular pathways for therapeutic intervention and better TNBC prognostication.

Although targeting metabolic dependencies emerges as an effective approach for the treatment of aggressive malignancies such as acute leukemia^5^, TNBC cells are highly heterogeneous in terms of their dependence on specific metabolic pathways^6^. Early studies indicated that TNBC cells utilize predominantly aerobic glycolysis instead of oxidative phosphorylation (OXPHOS) for energy production^7^. A recent study, however, shows that TNBC cells shift from glycolysis to mitochondrial OXPHOS during metastatic spread^8^. Similarly, upregulated fatty acid oxidation is detected in metastatic breast cancer^9^. Despite these findings, the dependence of TNBC cells on the tricarboxylic acid (TCA) cycle remains unclear.

Inside the TCA cycle, dihydrolipoamide S-succinyltransferase (DLST) is the E2 transferase of α-ketoglutarate dehydrogenase complex (KGDHC) that regulates the irreversible conversion of α-ketoglutarate to succinyl-CoA^10^. Here we show that high *DLST* expression predicts poor overall and recurrence-free survival among TNBC patients. DLST depletion impairs the growth, survival, and migration of TNBC cells that have relatively intact TCA-cycle function, while minimally affecting TNBC cells with the defective TCA cycle function. Importantly, DLST-dependent TNBC cells exhibit decreased tumor burden and invasion in zebrafish xenografts when treated with CPI-613, a lipoate derivative that can inhibit KGDHC activity and is currently in clinical trials for treating other cancers (NCT03793140; NCT03699319; NCT04217317; and NCT04593758). Together, our data demonstrate that DLST-dependence in TNBC dictates their usage of the TCA cycle to promote tumor cell growth and invasion. Our studies provide mechanistic insights for metabolic heterogeneity of TNBC and therapeutic implications for the DLST-dependent subsets.

## Results

### High *DLST* expression predicts poor overall and recurrence-free survival among TNBC patients

**Fig. 1:**
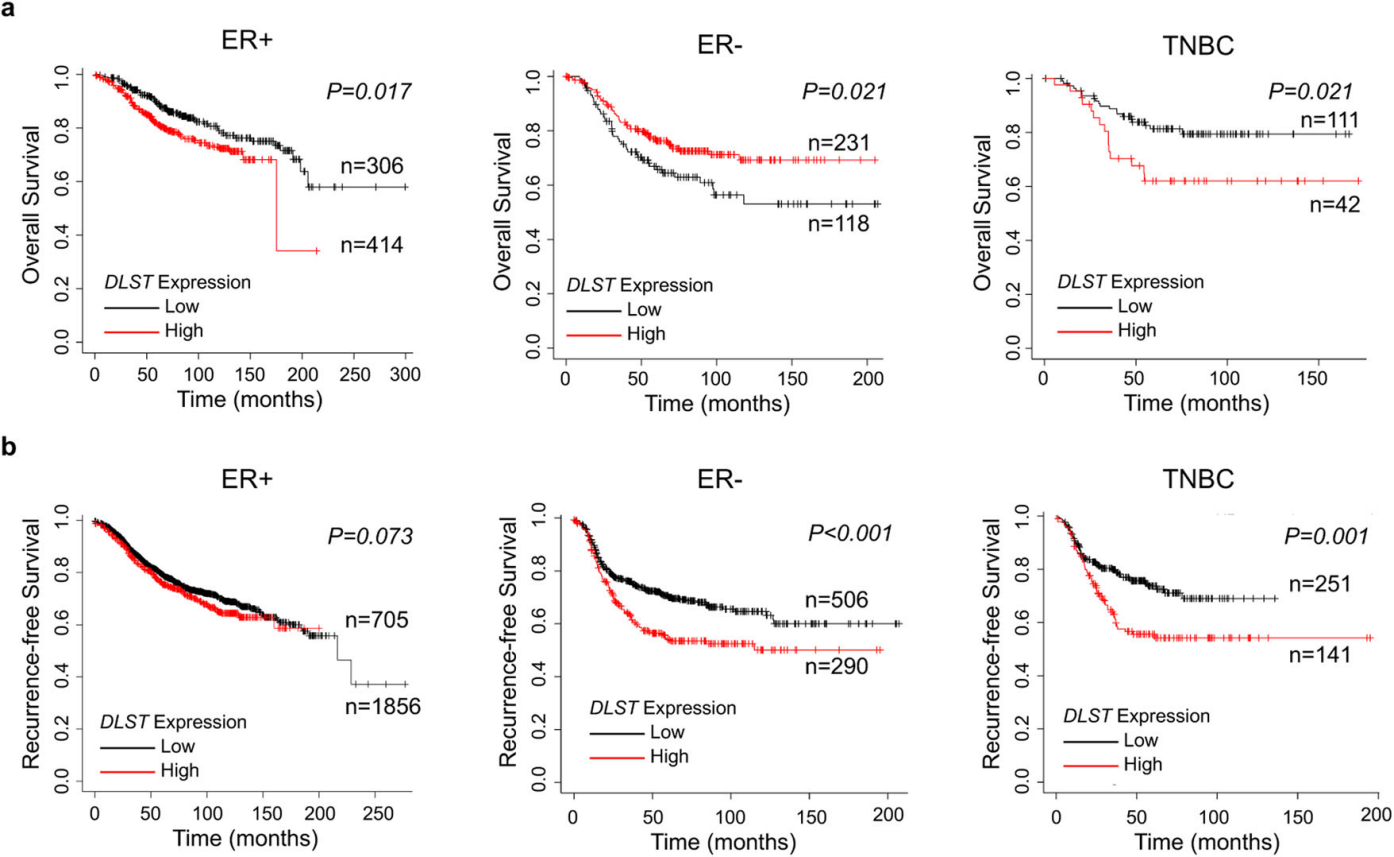
High *DLST* expression is associated with poor overall and recurrence-free survival in TNBC patients. **a**, **b** In silico Kaplan–Meier analysis of the breast cancer patients (http://kmplot.com/analysis/). The curves demonstrate the association of *DLST* expression with overall (**a**) and recurrence-free (**b**) survival in patients over time. Overall survival: *n* = 720 for ER+, *n* = 349 for ER−, and *n* = 153 for TNBC; and recurrence-free survival: *n* = 2561 for ER+, *n* = 796 for ER−, and *n* = 392 for TNBC. Patient samples were stratified using the best cut-off and compared with the log-rank test for statistical significance between curves.

We previously reported that DLST contributes to MYC-driven leukemogenesis^11^. To study the role of DLST in breast cancer pathogenesis, we performed in silico Kaplan–Meier survival analysis using an online tool (http://kmplot.com/analysis/)^12^. Patients were categorized into three groups based on the receptor’s expression in tumor cells: ER+, ER−, and TNBC^12^. We then correlated the expression of *DLST* and the other two KGDHC subunits, dihydrolipoamide dehydrogenase (*DLD*) and oxoglutarate dehydrogenase (*OGDH*), with survival probability among each patient group. We found that *DLST* expression is associated with poor overall survival with a similar trend for recurrence-free survival in the ER+ patients (Fig. 1a, b). Different from ER- patients, which showed inconsistent overall and recurrence-free survival, high *DLST* expression reliably predicted poor overall and recurrence-free survival among TNBC patients (Fig. 1a, b). A similar observation for recurrence-free survival was made for TNBC patients when analyzing another publicly available database (GSE2034)^13^ (Supplementary Fig. 1a), despite somewhat different criteria applied in defining receptor positivity compared to the KMplot dataset. Different from *DLST*, neither *DLD* nor OGDH expression could similarly predict TNBC patient prognosis in both datasets, although *OGDH* showed a trend as *DLST* in predicting TNBC patient prognosis (Supplementary Figs. 1–3). These findings demonstrate that high expression of *DLST* predicts disease relapse and poor survival among TNBC patients, indicating a different role of DLST in TNBC pathogenesis when compared to leukemia.

### Human TNBC cells exhibit differential dependence on DLST

**Fig. 2:**
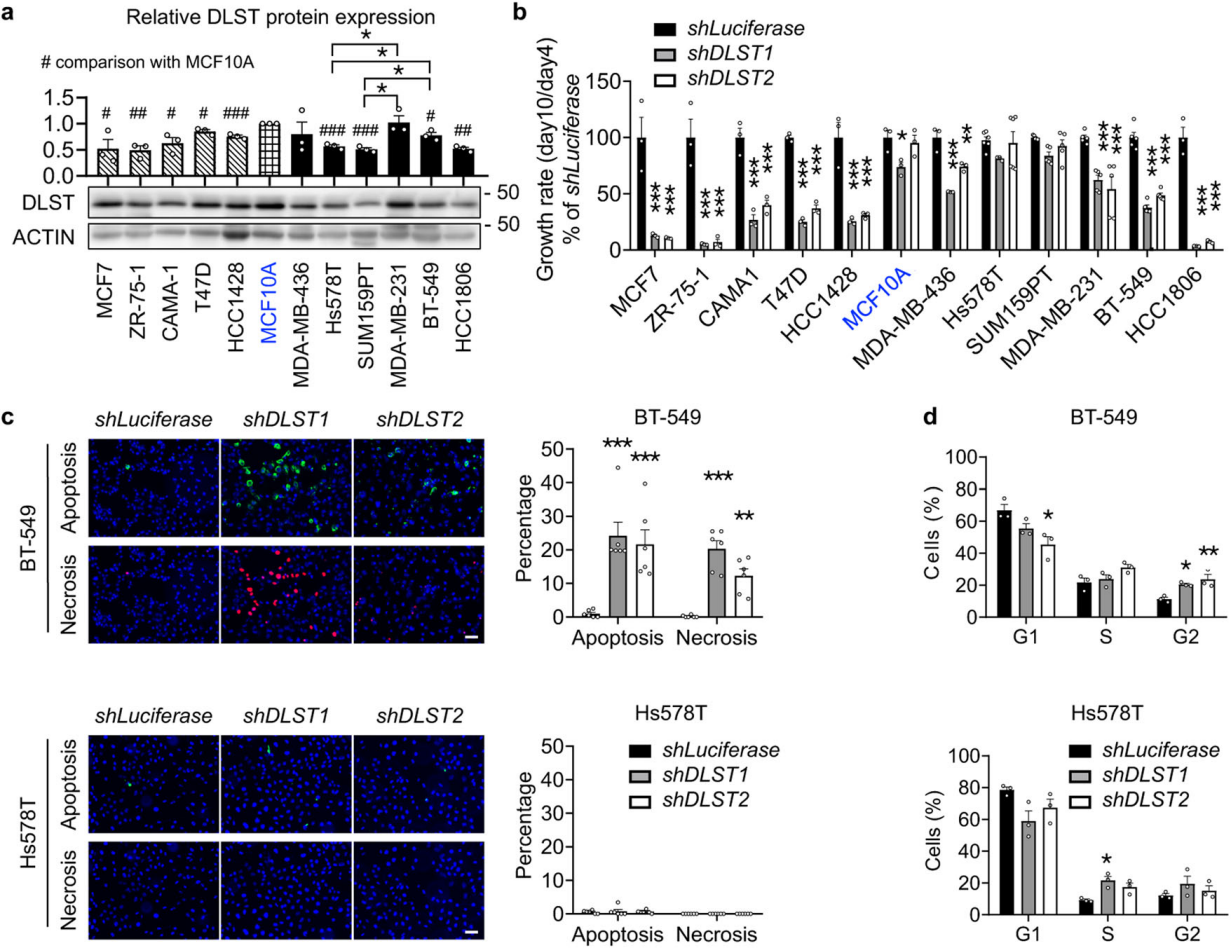
Human TNBC cell lines exhibit differential responses to DLST depletion. **a** Semi-quantitative western blotting analysis shows DLST expression in five human ER+ cell lines (striped bars), a non-transformed mammary gland MCF10A cell line (blue font), and six human TNBC cell lines (black bars), with ACTIN serving as the loading control (*n* = 3 of independent experiments). # in the bar figure indicates significant differences in DLST expression of breast cancer cell lines when compared to MCF10A cells. **b** Cell growth rates of cell lines in (**a**) transduced with *shLuciferase*, *shDLST1*, or *shDLST2* hairpin (*n* = 3–6 per group). The knockdown efficiency was validated by western blotting shown in Supplementary Fig. 5a, c. **c** Apoptosis and necrosis of TNBC cells were assessed at day 5 post-transduction by Annexin-V (green) and Ethidium homodimer III (red) staining, respectively, with DAPI (blue) staining for all cells. Representative images of BT-549 and Hs578T cells (left) together with their quantification (right) were shown (*n* = 6 per group). Scale bars = 20 µm. **d** Cell cycle distribution of BT-549 and Hs578T cells after *DLST* knockdown was shown as the percentage of cells in each cell-cycle phase (*n* = 3 biological samples). Data in (**a**–**d**) are presented as mean ± s.e.m. One-way analysis of variance (ANOVA) in (**b**, **d**) and an unpaired two-tailed *t*-test in (**a**, **c**) were used for statistical analyses. * or ^#^*P* ≤ 0.05, ** or ^##^*P* ≤ 0.01, *** or ^###^*P* ≤ 0.001.

Next, we utilized cell line models to study how DLST contributes to TNBC pathogenesis. Our western blotting analysis revealed that DLST protein expression fluctuates among breast cancer cell lines (Fig. 2a). Interestingly, DLST protein expression in all of the ER+ cell lines tested is lower than that of the non-transformed MCF10A cells (Fig. 2a). However, DLST expression in TNBC cell lines differs, with some having similar protein levels compared to the non-transformed MCF10A cells and others with lower expression of DLST, which mimics its protein and transcript levels in both TNBC patient samples and cell lines (Fig. 2a; Supplementary Fig. 4a–c). Among TNBC cell lines tested, Hs578T and SUM159PT were the only two that showed relatively lower DLST expression on both transcript and protein levels (Fig. 2a; Supplementary Fig. 4c). Next, we knocked down *DLST* by shRNA in breast cancer cell lines and found that all of the ER+ cell lines were sensitive to DLST depletion, while TNBC cell lines exhibited a differential dependence on DLST (Fig. 2b; Supplementary Fig. 5a, b). *DLST* knockdown significantly reduced the growth of several TNBC cell lines: HCC1806, BT-549, MDA-MB-231, and MDA-MB-436 (Fig. 2b; Supplementary Fig. 5b). However, DLST depletion minimally impacted SUM159PT and Hs578T TNBC cells, which had the lowest DLST expression, as well as the non-transformed MCF-10A cells (Fig. 2a, b; Supplementary Fig. 5a, b).

To understand the cellular mechanisms underlying TNBC’s differential response to DLST depletion, we analyzed DLST-dependent BT-549 and MDA-MB-231 cell lines, as well as independent Hs578T and SUM159PT cell lines. At 5 days post-transduction of *shLuciferase*, *shDLST1*, or *shDLST2* virus, we assessed the apoptosis and necrosis of these TNBC cells by measuring Annexin V and Ethidium homodimer III stainings. DLST depletion by shRNA significantly increased apoptosis and necrosis of BT-549 and MDA-MB-231 cells while minimally impacting Hs578T and SUM159PT cells (Fig. 2c; Supplementary Fig. 6a). Cell cycle analysis was then performed for these TNBC cells by propidium iodide staining and flow cytometry. DLST depletion caused a significant change in G1-phase and an increase in G2-phase arrest of BT-549 and MDA-MB-231 cells but only a moderate S or G1-phase change in Hs578T and SUM159PT cells (Fig. 2d; Supplementary Fig. 6b). Taken together, the above data support that subsets of the TNBC cell lines depend on DLST for growth and survival.

### Human DLST-dependent TNBC cells possess an intact TCA cycle function and utilize glutamine anaplerosis

**Fig. 3:**
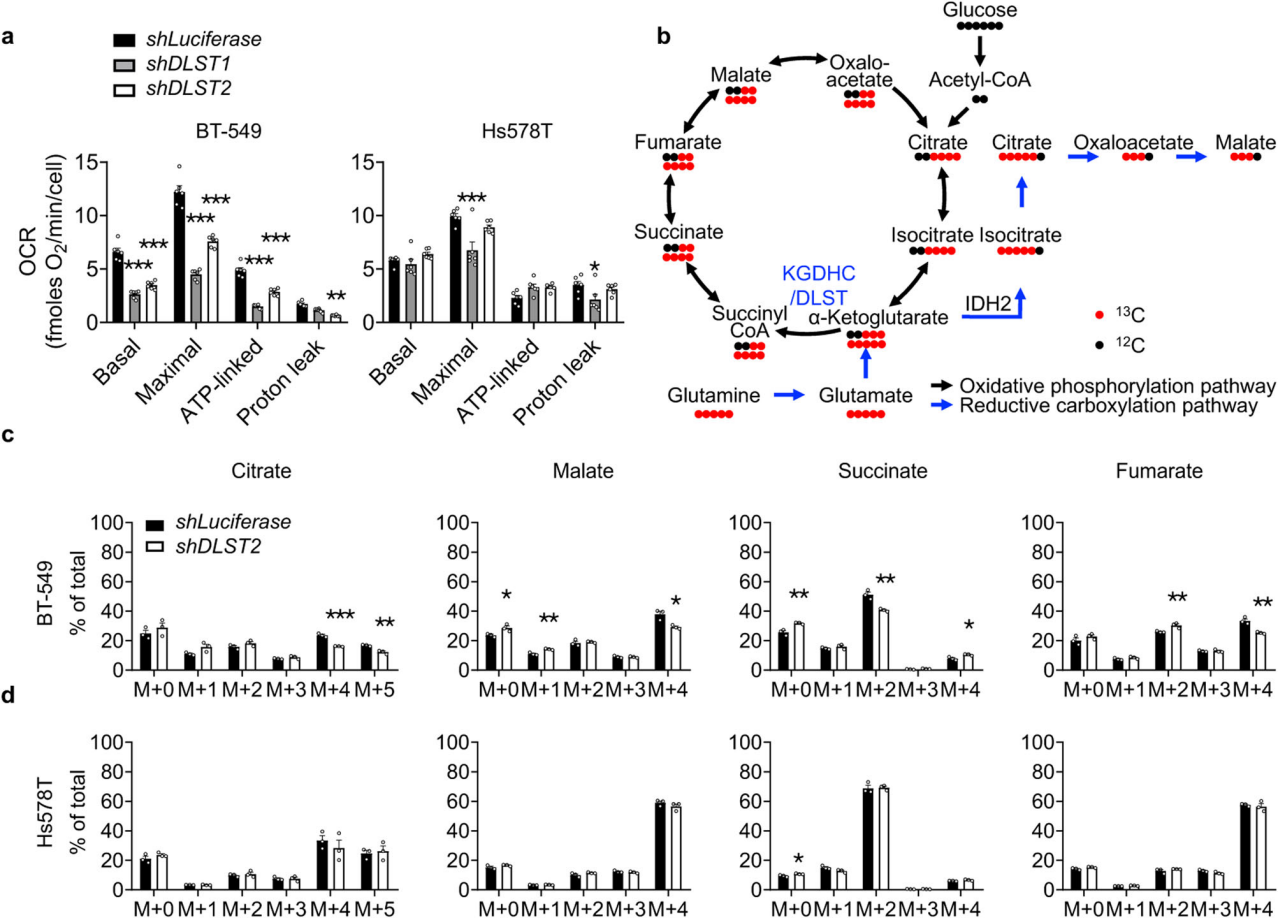
*DLST* knockdown decreases oxygen consumption rates and glutamine anaplerosis in DLST-dependent TNBC cells but not in independent ones. **a** Quantification of oxygen consumption rates (OCR) in BT-549 and Hs578T cells after *DLST* knockdown, assessed through Seahorse technology (*n* = 6). A representative OCR curve for each cell line was included in (Supplementary Fig. 7). **b** Scheme of ^12^C and ^13^C labeled glutamine contribution to TCA-cycle metabolites. **c**, **d** Percentage of ^13^C labeled glutamine-derived cycle metabolites was measured upon *DLST* knockdown in BT-549 (**c**) and Hs578T cells (**d**) (*n* = 3). Data are presented as mean ± s.e.m, and an unpaired two-tailed *t*-test was used for statistical analysis in (**a**, **c**, **d**). *P ≤ 0.05, **P ≤ 0.01, ***P ≤ 0.001.

Since DLST is a TCA cycle transferase^11^, we next evaluated the ability of these TNBC cell lines, BT-549, MDA-MB-231, SUM159PT, and Hs578T in utilizing the TCA cycle by galactose replacement assay. When galactose replaces glucose in the medium, cells will produce ATP preferentially through the TCA cycle instead of glycolysis^14,15^. Hence, if a cell can maintain its cellular ATP levels in the galactose medium, its TCA cycle function should be intact, and vice versa. Consistent with their dependence on DLST, BT-549 and MDA-MB-231 cells continuously produced ATP in the galactose medium with cellular ATP levels minimally affected (Supplementary Fig. 7). In contrast, SUM159PT and Hs578T, the two cell lines with lower dependency on DLST, experienced a significant decrease in ATP levels when cultured in the galactose medium (Supplementary Fig. 7). Next, we measured the oxygen consumption rates (OCR) by seahorse technology to further document the differential utilization of the TCA cycle in BT-549 and Hs578T cells. DLST depletion significantly decreased all aspects of OCR in DLST-dependent BT-549 cells, including basal, maximal, ATP-linked, and proton-leak OCR (Fig. 3a and Supplementary Fig. 8a). However, OCR is minimally impacted in DLST-independent Hs578T cells upon *DLST* knockdown (Fig. 3a and Supplementary Fig. 8a).

Given that TNBC cells are addicted to glutamine and that DLST/KGDHC governs the entry of glutamine into the TCA cycle^16–20^, we asked whether TNBC cells utilize glutamine anaplerosis. All TNBC cell lines tested are sensitive to glutamine withdrawal; however, only DLST-dependent TNBC cells can be partially rescued by the addition of exogenous TCA-cycle intermediates, dimethyl-2-oxoglutarate or mono-methyl hydrogen succinate (Supplementary Fig. 8b). Next, we examined the effect of DLST depletion on glutamine anaplerosis in both DLST-dependent and independent TNBC cells. Using ^13^C-labeled glutamine as a tracer, we measured the percentage changes of glutamine-derived metabolites upon *DLST* knockdown in DLST-dependent BT-549 and independent Hs578T cells (Fig. 3b). Both cell lines incorporated over 90% of the labeled glutamine in the presence and absence of *DLST* knockdown (Supplementary Fig. 9a). In addition, over 80% of α-ketoglutarate were labeled with ^13^C-glutamine carbons in both cell lines without significant differences between control and *DLST* knockdown cells (Supplementary Fig. 9b). These results indicate that ^13^C-labeled glutamine was incorporated into these cells efficiently and the α-ketoglutarate carbons were mainly from glutamine. Despite similar glutamine contribution to the TCA cycle, *DLST* knockdown in BT-549 cells significantly decreased the percentage of M+4 and M + 5 citrate, M+2 succinate, M+4 fumarate, and M+4 malate, while increasing the percentage of M+0/M+4 succinate, M+0/M+1 malate, and M+2 fumarate (Fig. 3c). In contrast, *DLST* knockdown had minimal effect on the contribution of glutamine to cycle intermediates in Hs578T cells (Fig. 3d). Collectively, these results demonstrate that the TNBC cell lines that depend on DLST for growth and survival have a higher capacity to utilize the TCA cycle, glutamine anaplerosis in particular, compared to the independent ones.

### *DLST* knockdown induces discrete metabolic alterations in human DLST-dependent TNBC cells

**Fig. 4:**
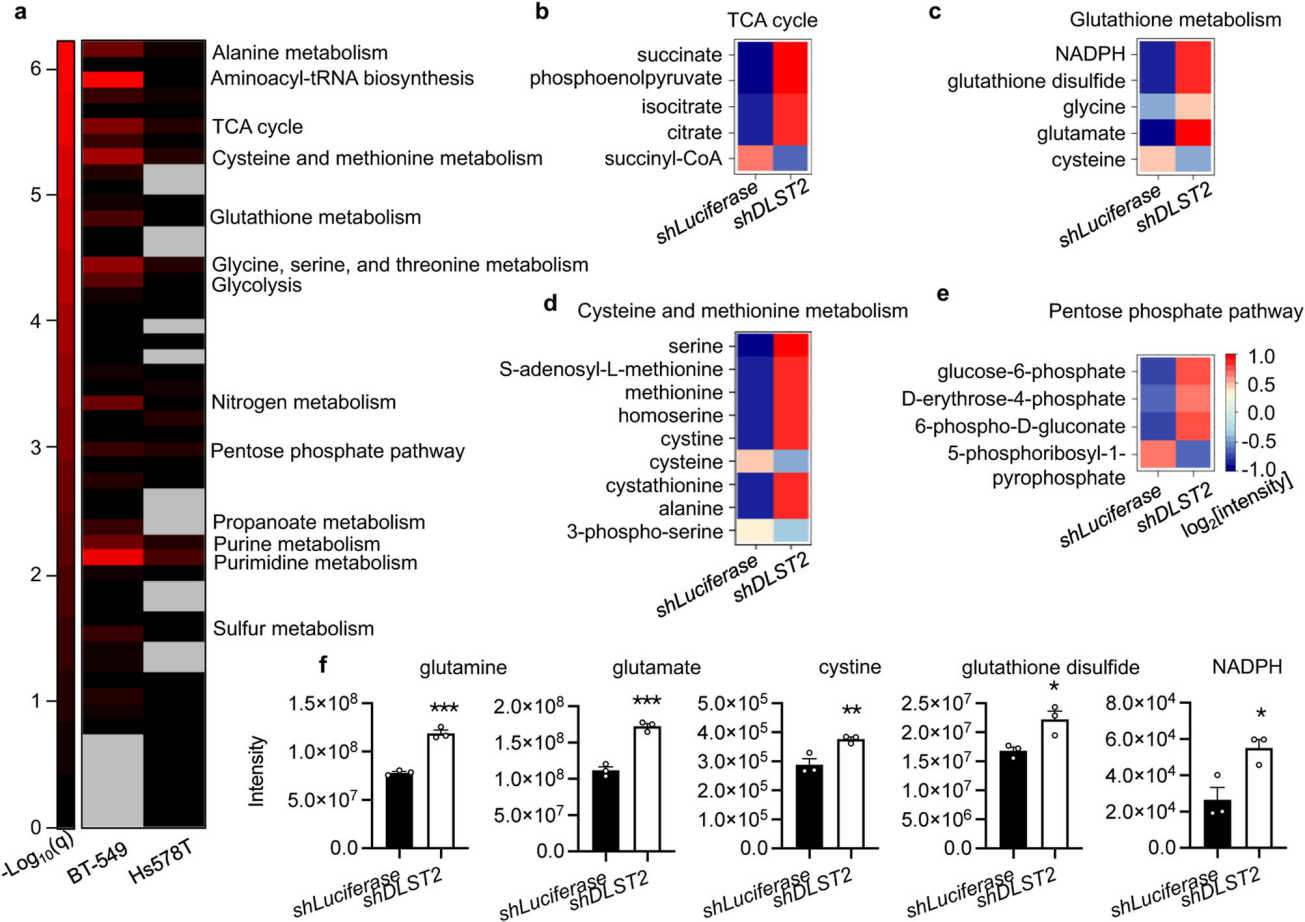
*DLST* knockdown significantly alters multiple metabolic pathways in DLST-dependent TNBC cells while minimally affecting independent ones. **a** Pathway enrichment analysis of unlabeled metabolomics profiling data in BT-549 and Hs578T cells. Missing values were represented by gray color. The enrichment score is represented as −log10(*q*-value). **b**–**e** The log2 values of normalized metabolite intensity in the pathways indicated are shown as the heatmap for BT549 cells (*n* = 3 independent experiments): TCA cycle (**b**), glutathione metabolism (**c**), cysteine and methionine metabolism (**d**), and pentose phosphate pathway (**e**). **f** Representative metabolites related to redox balance are shown in BT-549 cells after *DLST* knockdown (*n* = 3). Data are presented as mean ± s.e.m, and an unpaired two-tailed *t*-test was used for statistical comparison. *P ≤ 0.05, **P ≤ 0.01, ***P ≤ 0.001.

Next, we performed unbiased metabolomics profiling to assess the broad metabolic changes induced by *DLST* inactivation in human TNBC cells. Specifically, BT-549 and Hs578T cells were transduced with the *shLuciferase* or *shDLST* hairpin. At early day 4 post-transduction when we could not detect apparent cellular changes, total metabolites were extracted from these TNBC cells for mass spectrometry analysis as described^21^. Pathway enrichment analysis revealed that *DLST* inactivation significantly impacted multiple metabolic pathways in DLST-dependent BT-549 cells while minimally impacting DLST-independent Hs578T cells (Fig. 4a). Among those altered pathways, prominent changes were found in aminoacyl-tRNA biosynthesis, pyrimidine metabolism, and amino-acid metabolism pathways (Fig. 4a). As expected, the TCA cycle was significantly altered in BT-549 cells, with decreased succinyl-CoA and significantly increased succinate, phosphoenolpyruvate, isocitrate, and citrate levels (Fig. 4b; Supplementary Fig. 10a).

Besides the changes observed in the TCA cycle, several pathways regulating redox balance, such as glutathione metabolism, cysteine, and methionine metabolism, and the pentose phosphate pathway, were also significantly altered in BT-549 cells (Fig. 4b–e). *DLST* downregulation in BT-549 cells led to a significant increase of NADPH, glutathione disulfide, and glutamate levels, as well as a mild increase of glycine levels (Fig. 4c, f). However, cysteine showed a decrease under *DLST* knockdown (Fig. 4c, d), accompanied by apparent changes of cysteine metabolism, with significantly increased levels of S-adenosyl-L-methionine, methionine, and cystine (Fig. 4d). The disturbance of the pentose phosphate pathway upon *DLST* knockdown led to a significant increase of glucose-6-phosphate, D-erythrose-4-phosphate, and 6-phospho-D-gluconate levels, as well as a decrease in 5-phosphoribosyl-1-pyrophosphate levels (Fig. 4e). In sharp contrast to changes in BT-549 cells, *DLST* knockdown only caused minimal metabolic changes in Hs578T cells (Fig. 4a; Supplementary Fig. 10a, b). Hence, DLST depletion induces differential metabolic alterations in human TNBC cells based on their dependency on DLST.

### DLST depletion induces ROS production in human DLST-dependent TNBC cells

**Fig. 5:**
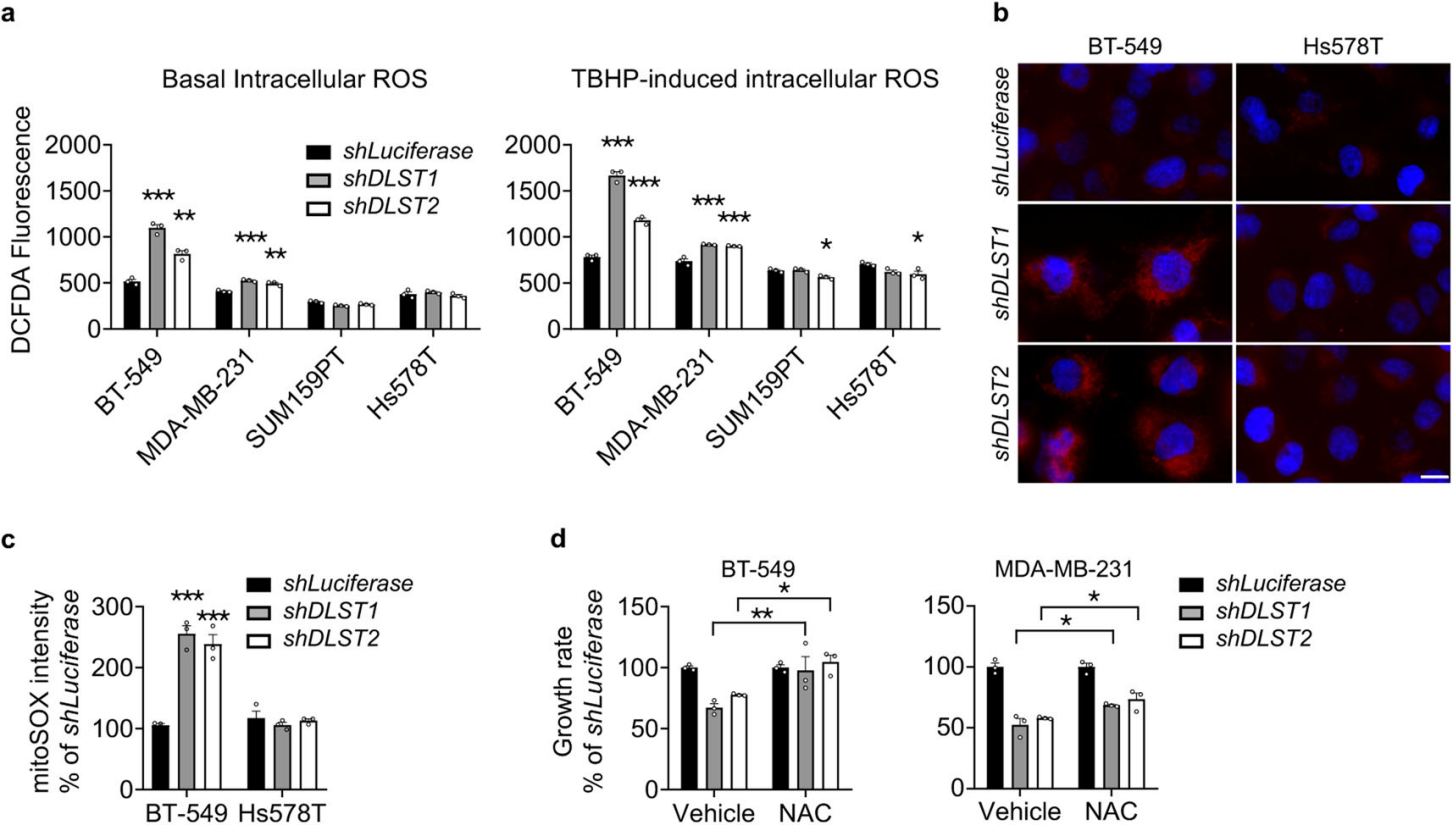
DLST depletion induces ROS production in DLST-dependent human TNBC cells. **a** In the late evening of day 4 post-transduction, ROS levels in four TNBC cell lines are measured using 2,7′-dichlorofluorescin diacetate (DCFDA) dye with (right) or without (left) TBHP treatment (*n* = 3; representative data from three independent experiments). Statistical differences were calculated using one-way ANOVA. **b**, **c** Mitochondrial ROS levels analyzed by mitoSOX staining in BT-549 and Hs578T cells in the presence or absence of *DLST* knockdown in the evening of day 4 post-transduction. Representative images of mitoSOX staining (**b**; red overlaid with DAPI staining) and image quantification (**c**) are shown (*n* = 3; representative data from three independent experiments). Scale bar = 10 µm. Statistical differences were calculated using two-way ANOVA. **d** Treatment with a ROS scavenger, NAC, restores the growth of BT-549 cells (left) while moderately rescues the growth of MDA-MB-231 cells (right) in the presence of *DLST* inactivation (*n* = 3; representative data from three independent experiments). Statistical differences were calculated using two-way ANOVA. Data in (**a**, **c**, **d)** are presented as mean ± s.e.m. *P ≤ 0.05, **P ≤ 0.01, ***P ≤ 0.001.

Since the metabolomics data revealed pathway alterations related to redox balance in BT-549 cells, we asked how *DLST* knockdown influenced the production of ROS in human TNBC cell lines, in the presence or absence of a ROS inducer, tert-butyl hydroperoxide (TBHP). *DLST* knockdown significantly increased the ROS production in DLST-dependent BT-549 and MDA-MB-231 cells but not in the independent SUM159PT and Hs578T cells (Fig. 5a). To assess the source of ROS production upon *DLST* knockdown, we stained BT-549 and Hs578T cells with a mitochondrial superoxidase (mitoSOX) dye. *DLST* inactivation induced a significant increase of mitoSOX signals in BT-549 cells but not in Hs578T cells, indicating mitochondria as the location of ROS production (Fig. 5b, c). To understand whether the increased ROS levels in DLST-dependent TNBC cells contributed to decreased cell growth upon *DLST* inactivation, we supplemented the media of TNBC cell lines with 2 mM of N-acetyl-L-cysteine (NAC) starting at 2 days post-transduction. Supplement with NAC partially rescued the growth of BT-549 and MDA-MB-231 cells upon *DLST* inactivation (Fig. 5d). These findings indicate that mitochondrial ROS production serves as one mechanism to impair cell growth upon DLST depletion in TNBC cells.

### DLST depletion inhibits the migration, growth, and invasion of human DLST-dependent TNBC cells

**Fig. 6:**
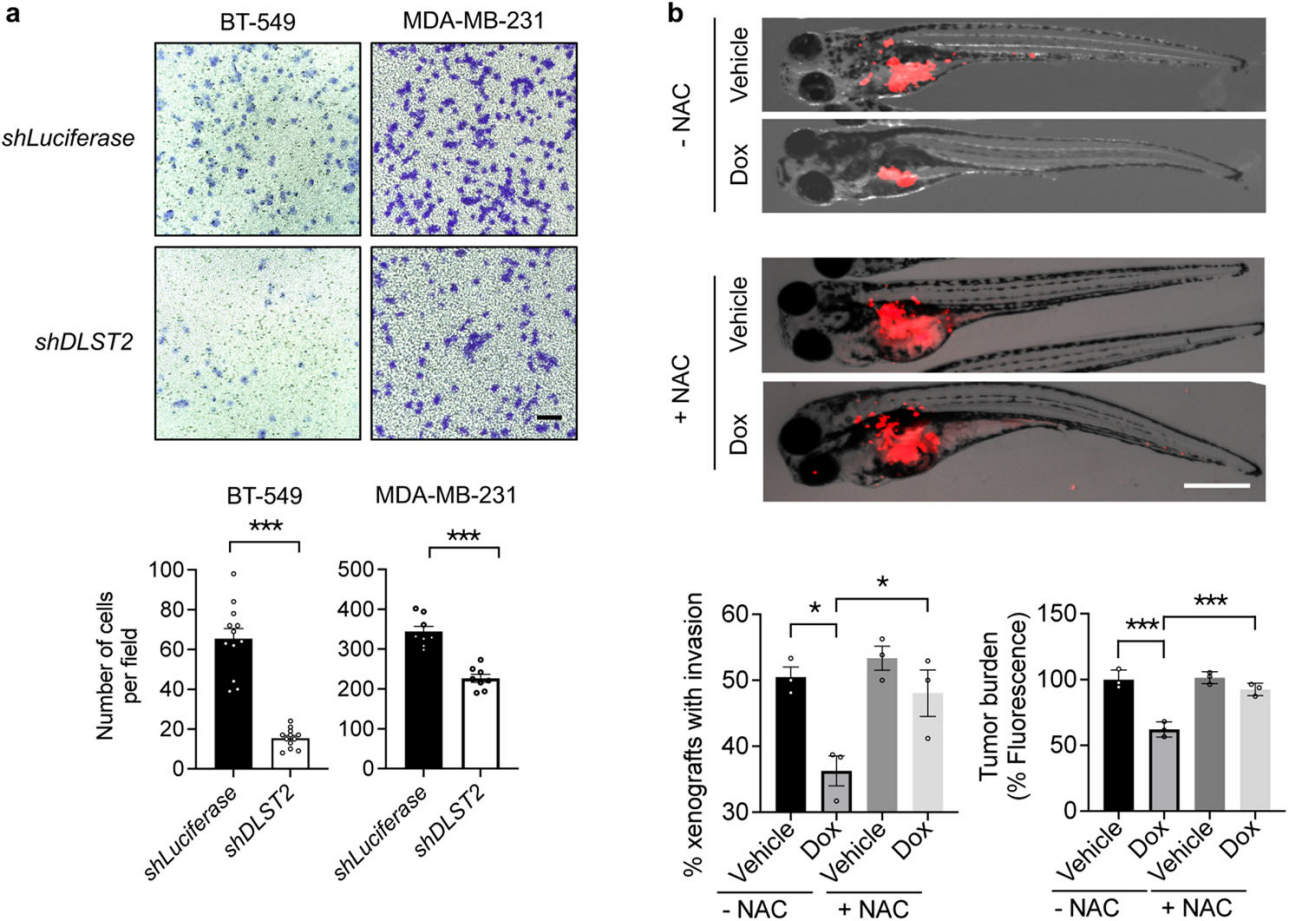
DLST depletion reduces migration, tumor burden, and invasion of human DLST-dependent TNBC cells. **a** The representative images (top) and quantitative data (bottom) of transwell cell migration assays for BT-549 and MDA-MB-231 cells in the presence or absence of *DLST* knockdown: 8–13 individual images from triplicated experiments were quantified for each group. Scale bar = 20 μm. **b** Overlay of brightfield and RFP channel images of zebrafish embryos transplanted with RFP+ BT-549 cells at 4 days of post-transplantation with or without NAC treatment (top panels). Quantification of zebrafish xenografts with tumor cell invasion (bottom, left) and burden (bottom, right): the average data from each experimental triplicate (23–29 fish per group) were presented as an individual dot. Tumor cell invasion was determined for the percentage of zebrafish xenografts that have tumor cells migrated away from the original injection site (bottom, left). Tumor burden was quantified based on the fluorescence intensity using ImageJ 1.52a (bottom, right). Scale bar = 0.5 mm. Data in (**a**, **b**) presented as mean ± s.e.m. *P ≤ 0.05, ***P ≤ 0.001. Statistical differences were calculated using one-way ANOVA.

**Fig. 7:**
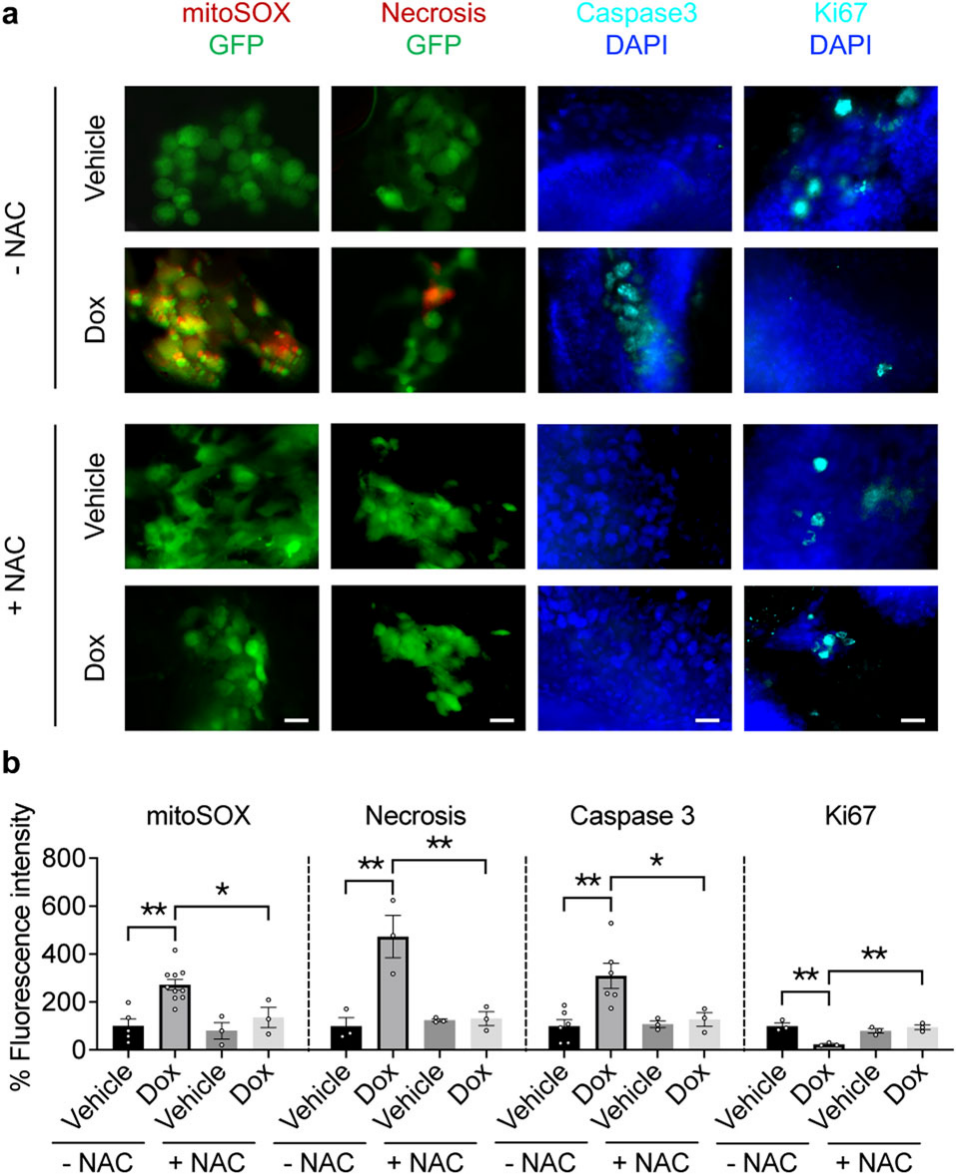
DLST depletion induces ROS, apoptosis, and necrosis while slowing the proliferation of human DLST-dependent TNBC cells, which can be rescued by NAC treatment. **a**, **b** Representative images (**a**) and quantitation (**b**) of mitoSOX (red, a marker for mitochondrial ROS), ethidium homodimer III (red, a marker for necrosis), active-caspase 3 (blue, a marker for apoptosis), and Ki-67 (blue, a marker for cell proliferation) stainings, which were overlaid either with GFP or DAPI signals of tumor cells in the presence or absence of NAC treatment (*n* = 3–10 fish per group). Scale bars = 20 μm. Data in (**b**) presented as mean ± s.e.m. *P ≤ 0.05, **P ≤ 0.01, ***P ≤ 0.001. Statistical differences were calculated using one-way ANOVA.

TNBC is an aggressive disease and often metastasizes to distant organs in patients^22^. To understand whether DLST plays a role in TNBC aggression, we performed in vitro transwell migration assays using human BT-549 and MDA-MB-231 cells. DLST depletion significantly slowed the migration of these TNBC cells (Fig. 6a). To assess the contribution of DLST to TNBC aggression in vivo, we transduced BT-549 cells with Doxycycline-inducible *shDLST2* and subsequently transplanted BT-549 cells into 2-day-old zebrafish, which lack adaptive immunity, to generate zebrafish xenografts. At 4 days of post-transplantation, DLST depletion significantly reduced tumor burden and invasion, which can be rescued by NAC treatment (Fig. 6b). Immunofluorescent staining of zebrafish xenografts revealed that DLST depletion induced mitochondrial ROS, necrosis, and apoptosis while reducing proliferation in BT-549 cells, all of which were again rescued by NAC treatment (Fig. 7a, b). The above data show that DLST supports the growth, survival, and invasion of TNBC cells that possess the intact TCA cycle function.

### DLST-dependent TNBC cells are sensitive to CPI-613 treatment

**Fig. 8:**
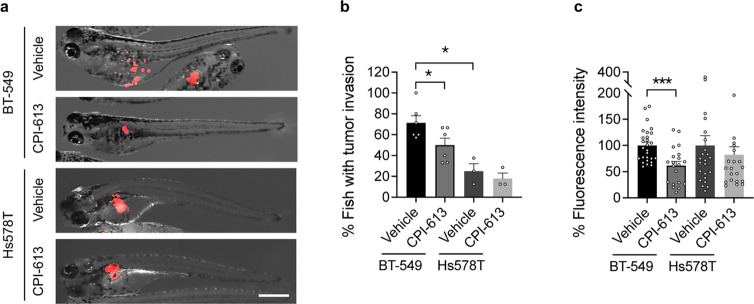
CPI-613 reduces tumor burden and invasion in zebrafish xenografts of DLST-dependent but not independent TNBC cells. **a** The representative overlay images of brightfield and RFP channel of zebrafish embryos transplanted with RFP+ BT-549 cells at 2 days of post-treatment. **b**, **c** Quantitative data indicate the changes of tumor invasion (**b**) and burden (**c**) after the treatment. Tumor cell invasion was determined for the percentage of zebrafish xenografts that have tumor cells migrated away from the original injection site in each experimental repeat (**a**, **b**): *n* = 3–6. Tumor burden was quantified based on the fluorescence intensity using ImageJ 1.52a (**a**, **c**). Fish number for BT-549: *n* = 27 for vehicle and 20 for CPI-613, respectively; and Hs578T: *n* = 24 for vehicle and 22 for CPI-613, respectively. Scale bar = 0.5 mm. Statistical difference was calculated using an unpaired two-tailed *t*-test. *P ≤ 0.05, ***P ≤ 0.001.

Since subsets of TNBC depend on DLST for growth and disease invasion, we next asked whether they are sensitive to compounds targeting DLST-mediated TCA functions. CPI-613 is a lipoate derivative that can inhibit KGDHC activity and is currently in clinical trials for treating relapsed/refractory lymphoma, metastatic pancreatic cancer, and clear cell sarcoma (NCT03793140; NCT03699319; NCT04217317; and NCT04593758). We thus applied CP-613 to treat zebrafish xenografts transplanted with human TNBC cells. We found that similar to the effect of DLST depletion, CPI-613 treatment effectively decreased tumor cell invasion in zebrafish xenografts transplanted with BT-549 cells (Fig. 8a, b). Consistent with their sensitivity to DLST depletion, CPI-613 treatment also reduced the tumor burden of these zebrafish xenografts (Fig. 8a, c). However, CPI-613 did not reduce the invasion or tumor burden of zebrafish xenografts transplanted with Hs578T cells, which are insensitive to DLST depletion (Fig. 8a–c). Interestingly, BT549 cells are more invasive in zebrafish xenografts compared to Hs578T cells (Fig. 8a, b). Hence, our data demonstrate that DLST-dependent TNBC cells are sensitive to the TCA-cycle inhibitor CPI-613.

## Discussion

TNBC is highly heterogeneous in its usage of metabolic pathways^6^. However, no viable markers exist to categorize TNBC based on their metabolic dependence. While TNBC has been considered glycolytic cancer with dysfunctional mitochondria^7,23,24^, recent studies indicate that chemotherapy-resistant TNBC and invasive cells from primary tumors utilize OXPHOS^25,26^. Despite this knowledge, whether TNBC utilizes the TCA cycle remains elusive. Here we performed *DLST* inactivation studies in a panel of TNBC cell lines to understand the metabolic heterogeneity and dependence on the TCA cycle. Our studies show that subsets of TNBC are dependent on DLST and the TCA cycle for growth and invasion. Galactose replacement and bioenergetic assays demonstrate that DLST-dependent TNBC cells utilize the TCA cycle and OXPHOS, while the less dependent TNBC cells minimally do. In DLST-dependent TNBC cells, DLST depletion inhibits OCR, leads to prominent alterations of TCA cycle metabolites, slows TNBC cell migration, and decreases disease burden and invasion in zebrafish xenografts. Hence, our studies demonstrate the differential utility of the TCA cycle in TNBC cells and unveil DLST-dependency as a valid molecular marker to predict TCA cycle usage in TNBC.

Glutamine is an important carbon source for TNBC^17^, but little is known about how glutamine is utilized in human TNBC cells. Our ^13^C labeled glutamine tracing data show that glutamine contributes to cycle intermediates in both DLST-dependent and independent TNBC cells. However, DLST depletion significantly reduces glutamine-contributed cycle intermediates in DLST-dependent TNBC cells, while minimally affecting independent ones. Consistent with our metabolomics data, DLST-dependent TNBC cells can be partially rescued upon glutamine withdrawal through supplementation of cycle intermediates, while the independent ones cannot. Hence, glutamine metabolism in TNBC is highly heterogeneous, and DLST/KGDHC mediates glutamine anaplerosis only in DLST-dependent TNBC cells. Interestingly, DLST depletion does not enhance the reductive carboxylation pathway, as no increased M+3 malate or M+5 citrate was detected in BT-549 or Hs578T cells. Instead, α-ketoglutarate in DLST-independent Hs578T cells can be converted into glutamate then glutamine to support cell survival under glutamine deprivation conditions^27^. On the other hand, DLST depletion in BT-549 cells leads to increased M+0/M+4 succinate, M+0/M+1 malate, and M+2 fumarate, indicating metabolic flexibility and compensatory effects. Our unbiased metabolomics profiling analysis reveals that *DLST* inactivation in BT-549 cells induces significant alterations in the TCA cycle, as well as nucleotide and amino-acid metabolic pathways, such as purine, pyrimidine, and serine and threonine metabolism. These data further support that glutamine anaplerosis replenishes cycle intermediates to support macromolecule and nucleotide synthesis in DLST-dependent TNBC cells.

The aggressive clinical course, dismal prognosis, and lack of effective targeted agents for TNBC render the identification of predictive biomarkers and the development of novel therapeutic approaches critical to improve patients’ survival. In particular, it is difficult to predict the sensitivity of chemotherapy or targeted therapy on TNBC patients, due to its intrinsic molecular and metabolic heterogeneity^28,29^. Our analysis of clinical data shows that high expression of DLST, a TCA cycle enzyme, is associated with an increased incidence of distant recurrence and poor survival among TNBC patients. Additionally, the majority of human DLST-dependent TNBC cell lines utilize the TCA cycle for growth and survival. Interestingly, TNBC cells with relatively high DLST expression use the TCA and are more invasive yet sensitive to DLST depletion, while most of those with relatively low DLST expression are not. Hence, our findings predict that TNBC patients with relatively high DLST expression in their tumor cells should be sensitive to inhibitors targeting the TCA cycle. CPI-613, an inhibitor targeting DLST/KGDHC in the TCA cycle, is in clinical trials for treating other types of cancers and shows promising safety and efficacy profiles (NCT03793140; NCT03699319; NCT04217317; and NCT04593758)^30,31^. Our data demonstrate that DLST-dependent TNBC cells are sensitive to CPI-613. Therefore, CPI-613 could serve as a targeted therapy for treating the aggressive TNBC with high DLST expression alone or in combination with chemotherapy.

## Methods

### Patient sample analysis

To assess the role of DLST in breast cancer pathogenesis, we performed in silico Kaplan–Meier analysis to determine the association of *DLST* expression with the overall and recurrence-free survival of breast cancer patients using the online tool and database (http://kmplot.com/analysis/)^12^. We applied the best cut-off, array, and immunohistochemistry (IHC) for ER, IHC for PR, and array for HER2 to stratify patients, which provides the optimal curve segregation. To further verify the findings, we re-analyzed the publicly available dataset (GSE2034) from NCBI/Genbank GEO database^13^. We also applied the best cut-off and receptor expression (ER < 1000, PR < 20, and HER2 < 3700 as being negative) to categorize patient samples into three categories: ER+ (*n* = 200), ER− (*n* = 86), and TNBC (*n* = 41). Cox regression analysis was utilized to determine the association of *DLST* expression with recurrence-free survival among breast cancer patients.

### Cell culture

All cell lines were obtained from the ATCC (www.atcc.org) and cultured at 37 °C supplemented with 5% CO_2_. Hs578T, MDA-MB-231, MDA-MB-436, SUM159PT, and HEK293T cells were cultured in DMEM medium (MT10013CV, Corning) supplemented with 10% fetal bovine serum (FBS, F0926, Sigma). BT-549, HCC1806, ZR-75-1, and HCC1428 cells were cultured in RPMI-1640 medium (MT10040CV, Corning) with 10% FBS. MCF10A cells were cultured at 37 °C with 5% CO_2_ in DMEM/F12 medium (SH30023.FS, Hyclone) containing 5% horse serum (26-050-088, Fisher Scientific), 20 ng ml^−1^ epidermal growth factor (E4127, Sigma), 100 ng ml^−1^ cholera toxin (C8052, Sigma), 10 ng ml^−1^ insulin (I9278, Sigma), and 500 ng ml^−1^ hydrocortisone (H4001, Sigma) as described^32^. T-47D cells were cultured in RPMI-1640 medium supplemented with 10% FBS and 0.2 U ml^−1^ bovine insulin (I0516, Sigma). CAMA-1 cells were cultured in EMEM medium (10009CV, Coring) with 10% FBS. MCF7 cells were cultured in EMEM medium with 10% FBS and 0.01 mg ml^−1^ human recombinant insulin (I9278, Sigma).

### Protein extraction and western blotting

Cells were lysed in RIPA buffer supplemented with 1× Halt proteinase inhibitor (87786, Thermo Scientific) and phosphatase inhibitor cocktail (BP479, Boston Bioproducts). Primary antibodies include anti-DLST (H00001743, Abnova) and anti-ACTIN (sc-47778, Santa Cruz Biotechnology). Secondary antibodies included goat anti-mouse (31430, Thermo Scientific) or anti-rabbit HRP antibodies (65-6120, Thermo Scientific). Chemiluminescence Supersignal West Pico was from Thermo Scientific (Cat# 34080). Autoradiographs were imaged using a G: BOX Chemi XT4 (Syngene).

### Lentivirus transduction and cell growth assay

The control and *DLST* shRNA hairpins (*shLuciferase*: 5′-CTTCGAAATGTCCGTTCGGTT-3′, *shDLST1*: 5′-CCCTAGTGCTGGTATACTATA-3′, and *shDLST2*: 5′-TGTCTCATAGCCTCGAATATC-3′) were cloned into the pLKO.1-puro vector. Lentivirus production and transduction were conducted as previously described^33^. The medium was changed 16 h (h) after virus transduction, with puromycin (1 μg ml^−1^, P7255, Sigma) added at 40 h post-transduction. On day 4 post-transduction, the cells were trypsinized and seeded into a 96-well plate at a density of 500 cells per well. CellTiter-Glo Luminescent Cell Viability Assay (G9242, Promega) was used to assess the rates of cell growth according to the manufacturer’s instruction on 4, 6, 8, and 10 days post-transduction. In the N-acetyl-L-cysteine (NAC, A7250, Sigma) rescue experiment, cell proliferation was observed at day 6 post-transduction.

### Cell cycle, apoptosis, and necrosis assay

Two million cells were fixed with 95% precooled ethanol, incubated overnight at −20 °C, washed with Dulbecco’s Phosphate Buffered Saline (DPBS, SH30028LS, Corning), and stained with propidium iodide/RNase staining buffer (BDB550825, BD Biosciences) containing 0.1% sodium citrate for 45 min (min) on ice in the dark. These cells were then analyzed by flow cytometry using BD LSRII SORP (BD Biosciences). ModFit LT software (Verify Software House) was used to generate DNA histograms. Each experiment was performed in triplicates.

The Apoptosis and Necrosis Quantification Kit (30017, Biotium) was used to quantify apoptotic and necrotic TNBC cells at 5 days post-transduction, according to the manufacture’s protocol. Images were taken using a Revolve microscope (ECHO) and data were analyzed by Image J software. Apoptotic and necrotic cells were counted and normalized relative to the total number of cells in each well.

### ROS detection

ROS detection was performed using 2,7′-dichlorofluorescin diacetate (DCFDA) according to the manufacturer’s instruction (ab113851, Abcam) with modifications. Briefly, on day 4 post-transduction, TNBC cells were trypsinized from 6-well plates and incubated with DCFDA (20 µM) with a cell density of 500,000 ml^−1^ for 45 min at 37 °C in the dark. These cells were then washed with DBPS, seeded into a 96-well plate at a density of 25,000 per well, and treated with or without 50 µM tert-butyl hydroperoxide (TBHP) for 16 h. The ROS signals were detected using a microplate reader (BMG LABTECH) under the excitation and emission wavelength at 485 nm and 535 nm, respectively.

### Metabolomics profiling and analysis

Glutamine-free DMEM or RPMI medium was supplemented with ^12^C or ^13^C isotope-labeled L-Glutamine (G8540 and 605166, Sigma) to prepare media for metabolomics experiments. Lentivirus transduction was performed as described above. On day 3 post-transduction, the medium was replaced with the 2 mM ^13^C or ^12^C glutamine medium for BT-549 cells and 4 mM ^13^C or ^12^C glutamine medium for Hs578T cells. 24 h later, cells were washed with DPBS twice and extracted for polar metabolites. Specifically, pre-chilled 80% methanol (MeOH, A412P-4, Fisher Scientific) at −80 °C was added to the cells. After 20 min of incubation at −80 °C, the resulting mixture was scraped, collected into a 15-ml centrifuge tube, and centrifuged at 4000 rpm for 5 min at 4 °C. Insoluble pellets were re-extracted with 1 ml of MeOH. The supernatants from two rounds of extraction were combined then dried with a Savant DNA 120 speedVac (Thermo Scientific). The unlabeled and ^13^C labeled metabolites were analyzed by LC-MS at the Beth Israel Deaconess Medical Center.

The cell number of each sample was used to normalize the raw value of polar metabolites, which were then visualized and analyzed for statistical differences in R v3.5.3. Metabolites with missing values in more than half of the samples were removed and the missing data for the retained metabolites were imputed with the half of minimum values of its corresponding metabolite. Metabolites with either *P* values less than 0.05 from a two-sided *t*-test or absolute fold change greater than 1.5 were selected for pathway enrichment analysis by MetaboAnalyst 4.0 (https://www.metaboanalyst.ca/). Isotope-labeled data were also processed similarly. FDR (false discovery rate) adjusted *P* values of pathway enrichment results for BT-549 and Hs578T cells with and without *DLST* knockdown were used to generate the pathway heatmap using the *heatmap.2* function in the R package *gplots*. Heatmap plots for metabolites in the chosen pathway were generated using the *levelplot* function in the R package *lattice*.

### Immunofluorescence staining and imaging

TNBC cells were seeded on the sterile cover glass and stained with mitoSOX^TM^ Red (M36008, Thermo Scientific), according to the manufacturing instructions. Cells were then fixed with 4% paraformaldehyde (158127, Sigma) for 10 min, permeabilized with DPBS containing 0.2% Triton X-100 (T8787, Sigma) for 5 min at room temperature, and co-stained with DAPI (300 nM, D3571, NEB). Cells were mounted using Fluoromount-G (0100-01, SouthernBiotech) on glass slides (12-550-15, Fisher Scientific). Images were captured using an RVL-100-G microscope (ECHO).

### Zebrafish xenograft assays

Zebrafish (*Danio rerio*) husbandry was performed as described^34^, in the aquatic facility at Boston University School of Medicine (BUSM), following the protocols approved by the Institutional Animal Use and Care Committee at BUSM.

Zebrafish embryos were gradually reared to 37 °C and injected with ~200 TNBC cells into the perivitelline space of each embryo at 2-day post-fertilization (dpf). BT-549 cells were transduced with doxycycline-regulated *shDLST2* and stained with a cell tracker dye (C7001, Thermo Fisher). At 3 h post-transplantation, zebrafish xenografts were sorted into four groups and incubated with fish water containing either vehicle (distilled water) or 10 μg ml^−1^ of doxycycline (D9891, Sigma) to induce *shDLST2* expression in the presence and absence of 300 μM NAC (A7250, Sigma). Fish water was refreshed daily. At day 2 post-transplantation, fish were observed under the microscope and their images were captured. For the CPI-613 treatment experiment, BT-549 and Hs578T cells transduced with pWPI-RFP were injected to fish embryos that were treated with 5 μM CPI-613 (HY-15453, MCE) or vehicle. On day 2 post-transplantation, zebrafish were euthanized and fixed with 4% Paraformaldehyde (158127, Thermo Scientific) overnight. The fixed fish were then imaged under the MVX10 microscope (OLYMPUS) or stored in 100% MeOH at −20 °C.

Fixed zebrafish embryos were rehydrated through series dilutions of ethanol (75%, 50%, and 25% in PBS) for 5 min each, permeabilized with DBST containing 1% Triton-100 for 4 × 15 min, and washed with DBST containing 0.2% Triton-100. Antibody blocking buffer (DBST containing 0.2% Triton-100, 10% horse serum, and 1% DMSO) was used to block the samples for 1 h at room temperature. Embryos were then incubated with anti-Ki67 (ab15580, Abcam) or anti-caspase-3 (559565, BD Biosciences) primary antibodies overnight at 4 °C, followed by incubation with the secondary antibody (anti-Cy5, A10523, Invitrogen) for 2 h at room temperature. Embryos were mounted in a glass dish and subjected to imaging using the RVL-100-G Microscope (ECHO).

### Statistics and reproducibility

The association of *DLST* expression with overall and recurrence-free survival among patients with different subtypes of breast cancer was assessed by Kaplan–Meier analysis. The comparison of the statistical difference between the survival curves was done with the log-rank test. The student’s *t*-test was used to analyze differences in DLST protein expression, OCR, ATP levels, metabolites, and isotope-labeled glutamine contribution to metabolic derivates for TNBC cells with control *vs. DLST* knockdown. One-way analysis of variance (ANOVA) was utilized to assess differences in cell growth rates, apoptosis, necrosis, cell cycle distribution, and ROS levels among TNBC cells in the presence or absence of *DLST* knockdown. Two-way ANOVA was used to analyze differences in mitoSOX staining, cell growth rates for NAC rescue experiments, tumor cell migration, tumor burden, and tumor invasion in zebrafish xenografts in the presence or absence of *DLST* knockdown. All experiments except re-analysis of the publicly available datasets were conducted at least three times independently. *P* values equal to or less than 0.05 were considered statistically significant without being adjusted for multiple comparisons.

### Reporting summary

Further information on research design is available in the Nature Research Reporting Summary linked to this article.

## Supplementary information

Supplementary Information

Description of Additional Supplementary Files

Supplementary Data

Reporting Summary

## Data Availability

We confirm that all relevant data and methods are included in the main Article and the Supplementary Information section. The source data for the graphs and charts in the figures is available as Supplementary Data file and any remaining information can be obtained from the corresponding author upon reasonable request.
