# Supplementary material for: DLST-dependence dictates metabolic heterogeneity in TCA-cycle usage among triple-negative breast cancer
Source: Commun Biol. 2021 Nov 16;4:1289. doi: 10.1038/s42003-021-02805-8 (PMC8595664; doi:10.1038/s42003-021-02805-8)
Supplement: Supplementary file 1 — Supplementary Information [file 42003_2021_2805_MOESM1_ESM.pdf]

Supplementary Information

Supplementary Methods

Patient sample analysis

In silico Kaplan-Meier analysis was performed to determine the association of *DLD* or *OGDH* expression with the overall and recurrence-free survival of breast cancer patients using the online tool and database (http://kmplot.com/analysis/)[1]. We applied array and immunohistochemistry (IHC) for ER, IHC for PR, and array for HER2 to stratify patients with the best cut-off for gene-of-interest expression, which provides the optimal curve segregation. To further verify the findings, we re-analyzed the publicly available dataset (GSE2034) from NCBI/Genbank GEO database[2]. We also applied the best cut-off and receptor expression (ER < 1000, PR < 20, and HER2 < 3700 as being negative) to categorize patient samples into three categories: ER+ (n=200), ER- (n=86), and TNBC (n=41). Cox regression analysis was utilized to determine the association of *DLST*, *DLD*, or *OGDH* expression with recurrence-free survival among breast cancer patients.

To compare *DLST* expression in normal tissues to different types of breast cancer patient samples, we obtained the dataset through the cBioPortal website (https://www.cbioportal.org/datasets)[3], and reanalyzed using *R studio* (3.5.3). There is a total of 2506 breast cancer samples, 1459 ER+, 444 ER-, and 217 TNBC with mRNA expression data. *DLST* mRNA expression is expressed as z-scores (relative to normal expression) from Illumina Human v3 microarray. Mean and standard deviation of expression z-scores and number of samples were used from three groups of patients to perform one-way ANOVA test.

To determine DLST protein expression among patient samples of different subtypes of breast cancer, we re-analyzed publicly available proteomics data [4]. There is a total of 122 breast

cancer samples: 82 for ER+; 39 for ER-; and 16 for TNBC. The protein expression is presented as z-scores (relative to normal expression). Mean and standard deviation of expression z-scores and number of samples were used from three groups of patients to perform one-way ANOVA test.

Galactose replacement assay

TNBC cells at the exponential growth phase were trypsinized, washed twice with DPBS, and resuspended in glucose-free DMEM or RPMI-1640 medium (11966025 or 11879020, Gibco), supplemented with 10% dialyzed FBS (26400044, Gibco) together with either 10 mM glucose or galactose (G8270 or G5388, Sigma). After cell density was adjusted to 30,000 ml^{-1}, 100 µl of cell mixture was seeded into a 96-well plate in triplicates. ATP contents were measured using the CellTiter-Glo kit (G9242, Promega) after 0, 2, 6, and 24 hours (h). Data were presented as the relative percentage of intracellular ATP contents normalized to the mean value in cells exposed to glucose at 0 h.

Mitochondrial bioenergetics profiling

Oxygen consumption rates (OCR) were measured using the XF96 Extracellular Flux analyzer (Seahorse Bioscience) as described[5]. Specifically, Hs578T and BT-549 cells were plated at a density of 10,000 or 18,000 cells per well on an XF96 plate, respectively. Cells were then incubated for 24 h in a humidified 37 °C incubator with 5% CO_2 in DMEM or RPMI medium. While sensor cartridges were calibrated, cell plates were rinsed and cultured in a commercial assay medium supplemented with 2 mM L-glutamine and then incubated in a 37°C non-CO_2 incubator for 1 h before the measurement. OCR was measured first under basal conditions, then in the presence of a complex V inhibitor oligomycin (2.5 µM, Sigma) to define ATP-coupled respiration,

or mitochondrial uncoupler FCCP (0.5 μM, Sigma) to assess maximal oxidative capacity, finally the combination of complex I inhibitor rotenone (2 μM, Sigma) and complex III inhibitor antimycin A (2 μM, Sigma) to eliminate mitochondrial respiration. Three measurements of OCR were obtained following the injection of each drug after optimization of conditions for each cell line. Total cell numbers in each well were determined by imaging the plate in a celigo imager and counting the cells under the brightfield fields.

Glutamine withdrawal and growth rescue with cycle intermediates

$3x10^3$ TNBC cells were seeded into each well of a 96-well plate in triplicates in glutamine-free DMEM or RPMI-1640 medium (10313021 or 42401018, Gibco), which is supplemented with 10% dialyzed FBS (26400044, Gibco), with or without 2 mM glutamine (G8540, Sigma). For the rescue experiment, 2 mM dimethyl 2-oxoglutarate (349631, Sigma) or mono-methyl hydrogen succinate (M81101, Sigma) were added into the wells with cells cultured without glutamine. At 48 h post treatment, cell viability were measured using the CellTiter-Blue® Cell Viability Assay reagent (G8080, Promega) following the manufacture's instruction. Data were normalized to the control cells with 2 mM glutamine in their media.

Quantitative real-time PCR (qRT-PCR)

Breast cancer cells and non-transformed MCF10A cells were cultured, harvested, and subjected to total RNA extraction using Trizol reagent (15596026, Invitrogen). cDNA was synthesized with a Reverse Transcription Kit (205311, Qiagen). SYBR Green PCR master mix (QP004, Genecopoeia) and a Step-One PCR instrument (Applied Biosystems) were utilized for the qRT-PCR reaction according to the manufacturer's instruction. The qRT-PCR primer

sequences include: *DLST*, forward 5'-GGTGGGAGAAAGCTGTTGGAGAC-3' and reverse 5'-GTGGAGTGCCTCCTTCGACTTTT-3'; β-ACTIN, forward 5'-GGATTCCTATGTGGGCGACG-3' and reverse 5'- ACATGATCTGGGTCATCTTCTCG-3'. All reactions were performed in triplicates.

Statistics and reproducibility

The association of *DLD* or *OGDH* expression with overall and recurrence-free survival among patients with different subtypes of breast cancer was assessed by Kaplan-Meier analysis. The comparison of the statistical difference between the survival curves was done with the log-rank test. The student's *t*-test was used to analyze differences in DLST protein and mRNA expression, ATP contents, metabolites, and isotope-labeled glutamine contribution to metabolic derivates for TNBC cells with control *vs.* *DLST* knockdown. One-way analysis of variance (ANOVA) was utilized to assess differences in cell growth rates among TNBC cells in the presence or absence of *DLST* knockdown. All experiments except re-analysis of the publicly available datasets were conducted at least three times independently. *P* values equal to or less than 0.05 were considered statistically significant without adjusted for multiple comparisons.

Figure Legends

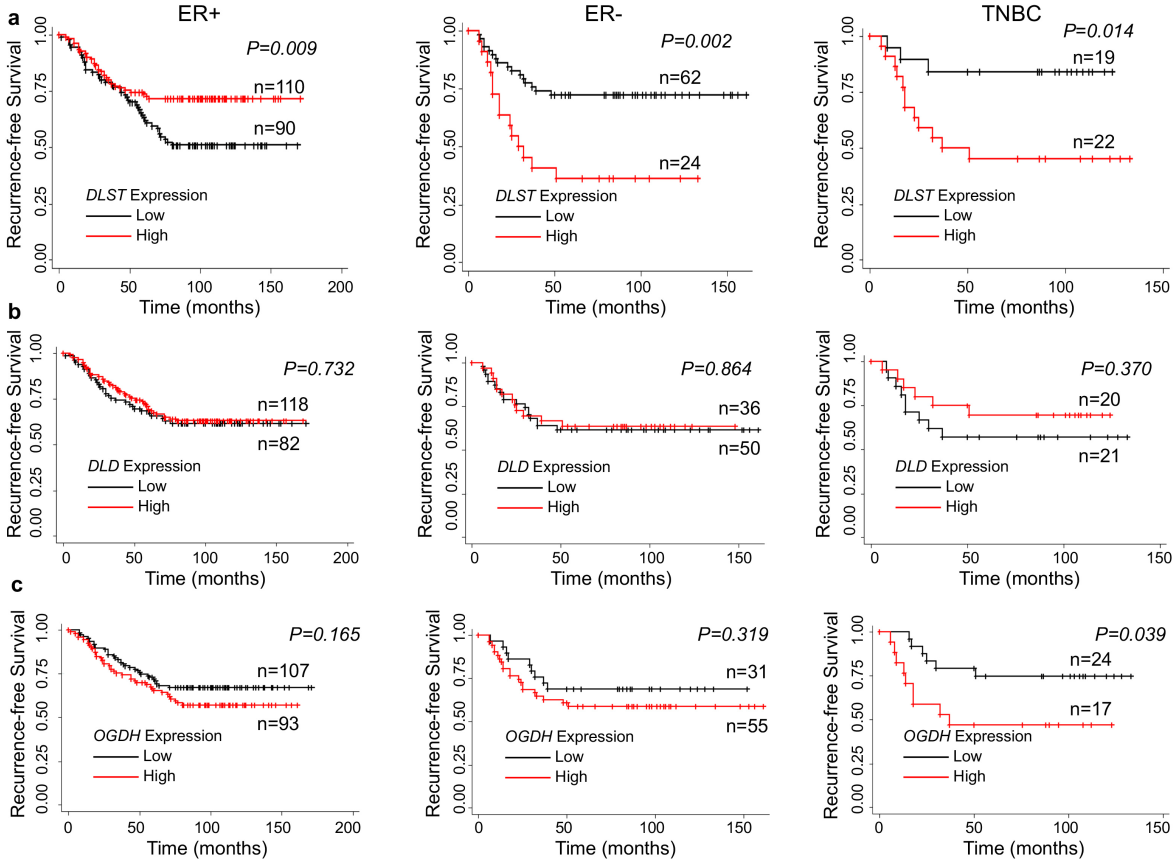

Supplementary Figure 1 | High *DLST* expression is associated with poor recurrence-free survival in TNBC patients. **a-c**, Kaplan-Meier re-analysis of published data for the association of *DLST* (**a**), *DLD* (**b**), and *OGDH* (**c**) expression with recurrence-free survival among breast cancer patients (GSE2034)[1]: ER+ breast cancer (n = 200), ER- breast cancer (n = 86), and TNBC (n = 41). The overall survival data is not available for this database. Patient samples were stratified using the best cut-off and compared with the log-rank test for statistical significance between curves.

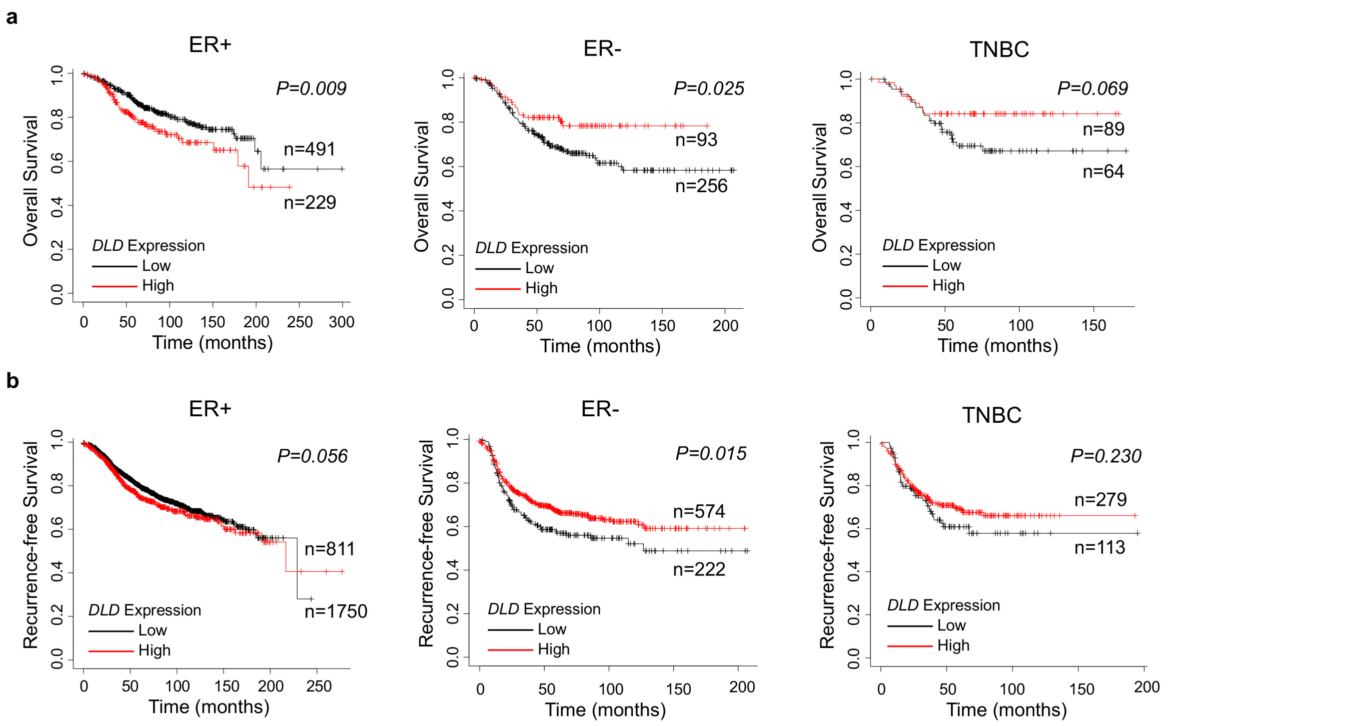

Supplementary Figure 2 | *DLD* expression does not predict overall or recurrence-free survival among TNBC patients. a-b, *in silico* Kaplan-Meier analysis of the association between *DLD* expression and overall (**a**) as well as recurrence-free (**b**) survival of breast cancer patients using an online tool (http://kmplot.com/analysis/). Overall survival: n = 720 for ER+, n = 349 for ER-, and n = 153 for TNBC; and recurrence-free survival: n = 2561 for ER+, n = 796 for ER-, and n = 392 for TNBC. Patient samples were stratified using the best cut-off and compared with the log-rank test for statistical significance between curves.

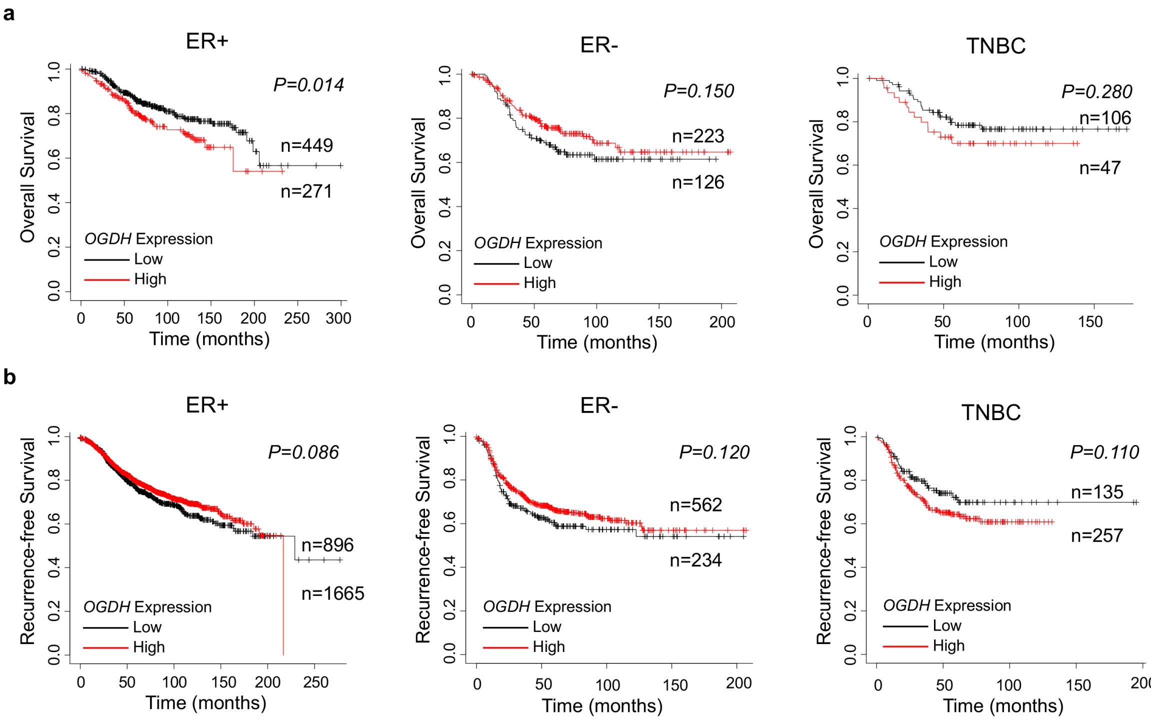

Supplementary Figure 3 | *OGDH* expression does not predict overall or recurrence-free survival among TNBC patients. a-b, *in silico* Kaplan-Meier analysis of the association between *OGDH* expression and overall (**a**) as well as recurrence-free (**b**) survival of breast cancer patients using an online tool (http://kmplot.com/analysis/). Overall survival: n = 720 for ER+, n = 349 for ER-, and n = 153 for TNBC; and recurrence-free survival: n = 2561 for ER+, n = 796 for ER-, and n = 392 for TNBC. Patient samples were stratified using the best cut-off and compared with the log-rank test for statistical significance between curves.

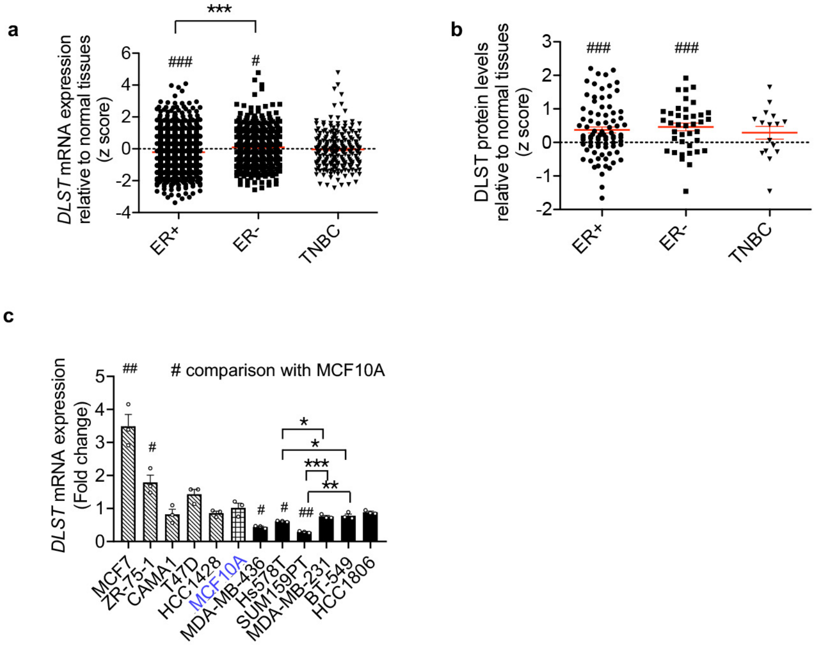

Supplementary Figure 4 | DLST levels in breast cancer patient samples and cell lines. a, *DLST* transcript levels in ER+, ER- and TNBC patient samples relative to normal tissues (data re-analyzed from a database through the cBioPortal website[3]). **b**, DLST protein levels in ER+, ER- and TNBC patient samples relative to normal tissues (data re-analyzed from a publicly available database[4]). **c**, *DLST* transcript levels in five human ER+ cell lines (HCC1428, T47D, CAMA-1, ZR-75-1, and MCF7: dashed bars), a non-transformed mammary gland MCF10A cell line (boxed bar), and six human TNBC cell lines (HCC1806, BT-549, MDA-MB-231, SUM159T, Hs578T, and MDA-MB-436: solid black bars). Although *DLST* mRNA expression appeared to be higher in our ER+ breast cancer cell lines than that in patient samples, this is likely due to the lack of data representation from the very limited number of cell lines. # indicates the significant difference among different subtypes of breast cancer patient samples or between breast cancer cell lines when compared to MCF10A cells. One-way ANOVA was used for statistical analyses in (**a-c**). * or $^{\#}$ $P \leq 0.05$, ** or $^{\#\#}$ $P \leq 0.01$, *** $P \leq 0.001$.

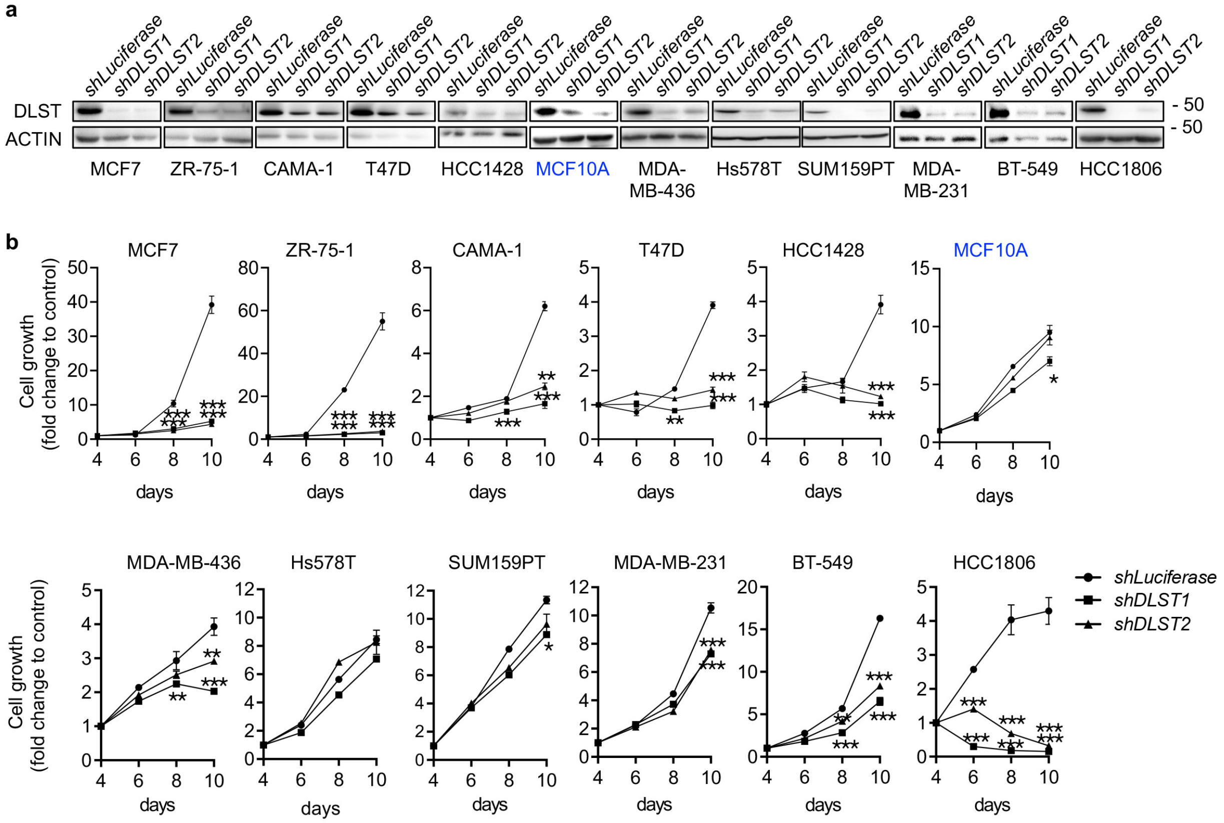

Supplementary Figure 5 | *DLST* inactivation differentially impacts the growth of breast cancer cell lines. a, Western blotting analysis of DLST protein levels at day 4 post-transduction of *shLuciferase*, *shDLST1,* or *shDLST2* hairpin in ER+, non-transformed MCF10A, and TNBC cell lines. **b**, Relative cell growth curve for each cell line upon *DLST* inactivation. Representative data from three independent experiments are presented as mean ± s.e.m in (**a-b**). * $P \leq 0.05$, ** $P \leq 0.01$, *** $P \leq 0.001$.

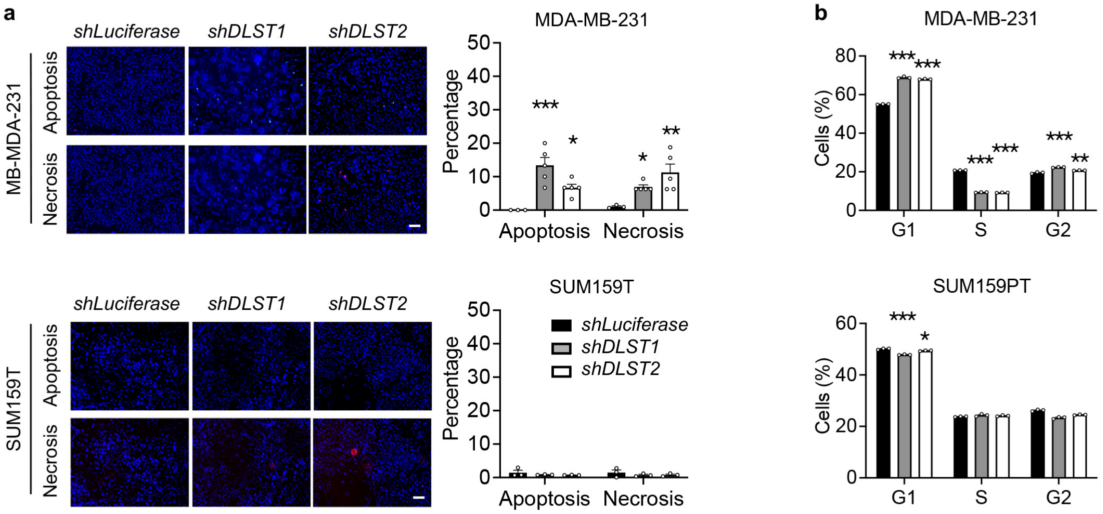

Supplementary Figure 6 | Human TNBC cell lines exhibit different dependence on DLST. a, Apoptosis and necrosis of TNBC cells were assessed at day 5 post-transduction by Annexin-V (green) and Ethidium homodimer III (red) staining, respectively, with DAPI (blue) staining for all cells. Representative images of MDA-MB-231 and SUM159PT cells (left) together with their quantification (right**)** were shown (n = 6 per group). Scale bars = 20 μm. **b**, Cell cycle distribution of MDA-MB-231 and SUM159PT cells after *DLST* knockdown was shown as the percentage of cells in each cell-cycle phase (n = 3 biological samples). Data in (**a,b)** are presented as mean ± s.e.m. One-way analysis of variance (ANOVA) in (**a**) and an unpaired two-tailed *t*-test in (**b**) were used for statistical analyses. * ≤ 0.05, ** $P \leq 0.01$, *** $P \leq 0.001$.

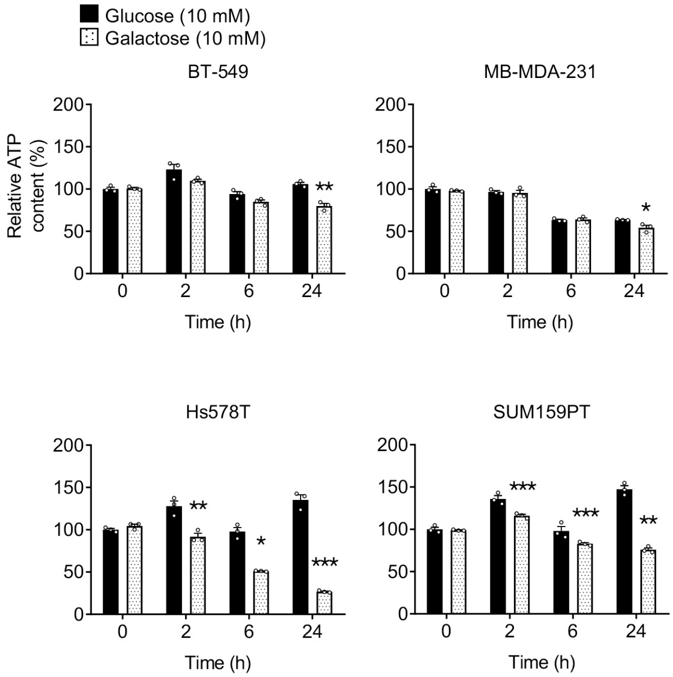

Supplementary Figure 7 | Human TNBC cell lines exhibit different capabilities in utilizing the TCA cycle. Relative ATP contents in BT-549, MDA-MB-231, Hs578T, and SUM159PT cells are shown at indicated time points in the presence of 10 mM glucose or galactose (n = 3). Representative data from three independent experiments are presented as mean ± s.e.m, with an unpaired two-tailed *t*-test to calculate statistical significance. * $P \leq 0.05$, ** $P \leq 0.01$, *** $P \leq 0.001$.

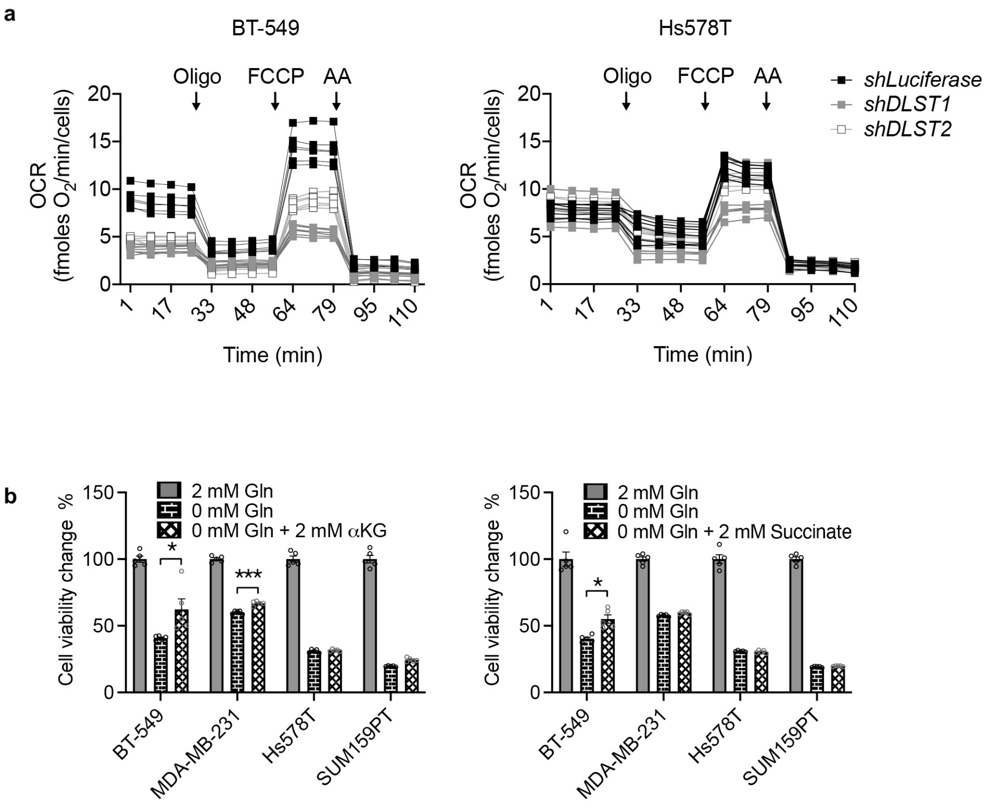

Supplementary Figure 8 | Differential usage of the TCA cycle and glutamine anaplerosis in human TNBC cells. a, Different types of OCR in BT-549 and Hs578T cells after *DLST* knockdown (n = 6), following the addition of oligomycin (Oligo, 2.5 μM), the uncoupler FCCP (0.5 μM), or the electron transport inhibitor rotenone and antimycin A (AA, 2 μM). **b,** Exogenous supplement of succinate and α-ketoglutarate can partially rescue the growth of DLST-dependent TNBC cells (BT-549 and MDA-MB-231) but not the independent ones (SUM159PT and Hs578T) upon glutamine withdrawal. Representative data from three independent experiments are presented as mean ± s.e.m, with a One-way ANOVA used for statistical analyses to calculate statistical significance. * $P \leq 0.05$, ** $P \leq 0.01$, *** $P \leq 0.001$.

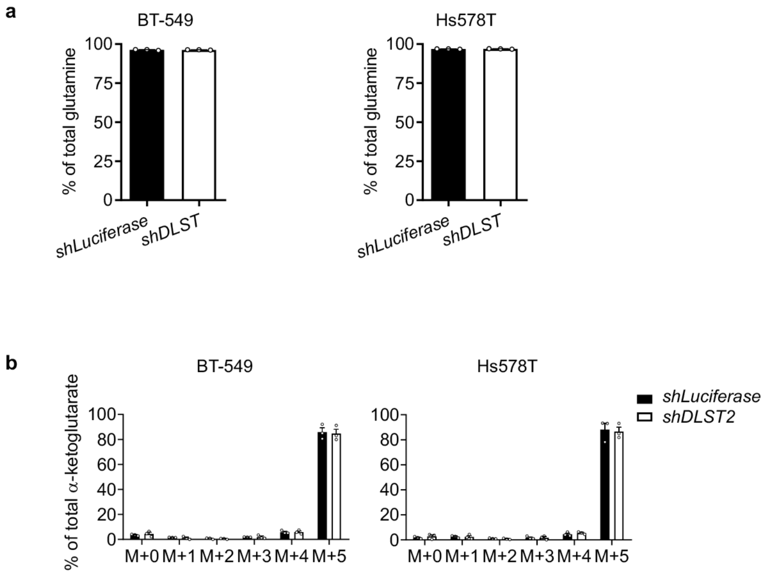

Supplementary Figure 9 | ^{13}C labeled glutamine is efficiently incorporated into TNBC cells. **a-b**, Percentage of ^{13}C labeled glutamine (**a**) or α-ketoglutarate (**b**) incorporated into BT-549 and Hs578T cells in the presence or absence of *DLST* knockdown (n = 3). Data are presented as mean ± s.e.m, and an unpaired two-tailed *t*-test was used to calculate statistical significance.

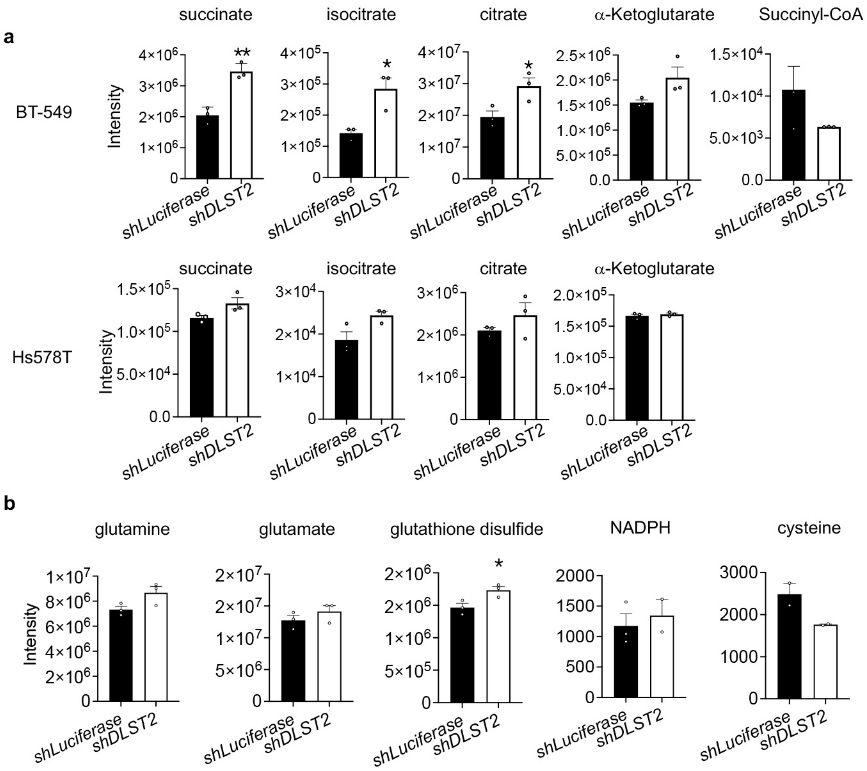

Supplementary Figure 10 | DLST depletion significantly alters metabolite levels in BT-549 but minimally in Hs578T cells. **a**, Changes of TCA-cycle metabolites – succinate, isocitrate, citrate, α-ketoglutarate, and succinyl-CoA – in BT-549 and Hs578T cells (n = 3). Succinyl-CoA was undetectable in Hs578T cells. **b**, Changes of glutamine, glutamate, glutathione disulfide, NADPH, and cysteine in Hs578T cells after *DLST* knockdown (n = 3). Cystine was undetectable. Data in (**a-b**) are presented as mean ± s.e.m, and an unpaired two-tailed *t*-test was used to calculate statistical significance. $*P \leq 0.05$, $** P \leq 0.01$.

Supplementary References

1. Gyorffy, B. *et al.* An online survival analysis tool to rapidly assess the effect of 22,277 genes on breast cancer prognosis using microarray data of 1,809 patients. *Breast cancer research and treatment* **123**, 725-731, doi:10.1007/s10549-009-0674-9 (2010).
2. Wang, Y. *et al.* Gene-expression profiles to predict distant metastasis of lymph-node-negative primary breast cancer. *Lancet (London, England)* **365**, 671-679, doi:10.1016/s0140-6736(05)17947-1 (2005).
3. Pereira, B. *et al.* The somatic mutation profiles of 2,433 breast cancers refines their genomic and transcriptomic landscapes. *Nat Commun* **7**, 11479, doi:10.1038/ncomms11479 (2016).
4. Krug, K. *et al.* Proteogenomic Landscape of Breast Cancer Tumorigenesis and Targeted Therapy. *Cell* **183**, 1436-1456 e1431, doi:10.1016/j.cell.2020.10.036 (2020).
5. Wu, M. *et al.* Multiparameter metabolic analysis reveals a close link between attenuated mitochondrial bioenergetic function and enhanced glycolysis dependency in human tumor cells. *American Journal of Physiology-Cell Physiology* **292**, C125-C136, doi:10.1152/ajpcell.00247.2006 (2007).
