# Supplementary material for: DLST-dependence dictates metabolic heterogeneity in TCA-cycle usage among triple-negative breast cancer
Source: Commun Biol. 2021 Nov 16;4:1289. doi: 10.1038/s42003-021-02805-8 (PMC8595664; doi:10.1038/s42003-021-02805-8)
Supplement: Supplementary file 2 — Description of Additional Supplementary Files [file 42003_2021_2805_MOESM2_ESM.pdf]

Description of Additional Supplementary Files

File name: Supplementary Data

Description: Source data for graphs and charts generated for the main figures and supplementary figures.
