# Supplementary material for: DLST-dependence dictates metabolic heterogeneity in TCA-cycle usage among triple-negative breast cancer
Source: Commun Biol. 2021 Nov 16;4:1289. doi: 10.1038/s42003-021-02805-8 (PMC8595664; doi:10.1038/s42003-021-02805-8)
Supplement: Supplementary file 4 — Reporting Summary [file 42003_2021_2805_MOESM4_ESM.pdf]

nature research

Corresponding author(s): Hui Feng

Last updated by author(s): Aug 14, 2021

Reporting Summary

Nature Research wishes to improve the reproducibility of the work that we publish. This form provides structure for consistency and transparency in reporting. For further information on Nature Research policies, see our Editorial Policies and the Editorial Policy Checklist.

Statistics

For all statistical analyses, confirm that the following items are present in the figure legend, table legend, main text, or Methods section.

n/a	Confirmed	
☐	☒	The exact sample size (*n*) for each experimental group/condition, given as a discrete number and unit of measurement
☐	☒	A statement on whether measurements were taken from distinct samples or whether the same sample was measured repeatedly
☐	☒	The statistical test(s) used AND whether they are one- or two-sided<br>*Only common tests should be described solely by name; describe more complex techniques in the Methods section.*
☒	☐	A description of all covariates tested
☐	☒	A description of any assumptions or corrections, such as tests of normality and adjustment for multiple comparisons
☐	☒	A full description of the statistical parameters including central tendency (e.g. means) or other basic estimates (e.g. regression coefficient) AND variation (e.g. standard deviation) or associated estimates of uncertainty (e.g. confidence intervals)
☒	☐	For null hypothesis testing, the test statistic (e.g. *F*, *t*, *r*) with confidence intervals, effect sizes, degrees of freedom and *P* value noted<br>*Give P values as exact values whenever suitable.*
☒	☐	For Bayesian analysis, information on the choice of priors and Markov chain Monte Carlo settings
☒	☐	For hierarchical and complex designs, identification of the appropriate level for tests and full reporting of outcomes
☒	☐	Estimates of effect sizes (e.g. Cohen's *d*, Pearson's *r*), indicating how they were calculated

Our web collection on statistics for biologists contains articles on many of the points above.

Software and code

Policy information about availability of computer code

Data collection	G: BOX Chemi XT4 (Syngene), BD LSRII SORP (BD Biosciences), LC-MS at Beth Israel Deaconess Medical Center, XF96 Extracellular Flux analyzer (Seahorse Bioscience), microplate reader (BMG LABTECH), Revolve microscope (ECHO), Step-One PCR instrument (Applied Biosystems)
Data analysis	ModFit LT software (Verify Software House), R v3.5.3., MetaboAnalyst 4.0 (https://www.metaboanalyst.ca/), Image J (Fiji), Microsoft Excel (Excel 365), and GraphPad Prism (v8.0)

For manuscripts utilizing custom algorithms or software that are central to the research but not yet described in published literature, software must be made available to editors and reviewers. We strongly encourage code deposition in a community repository (e.g. GitHub). See the Nature Research guidelines for submitting code & software for further information.

Data

Policy information about availability of data

All manuscripts must include a data availability statement. This statement should provide the following information, where applicable:

- Accession codes, unique identifiers, or web links for publicly available datasets
- A list of figures that have associated raw data
- A description of any restrictions on data availability

Data supporting the findings of this study are described in the article. The raw data are available from the corresponding author upon request.

Field-specific reporting

Please select the one below that is the best fit for your research. If you are not sure, read the appropriate sections before making your selection.

☒ Life sciences ☐ Behavioural & social sciences ☐ Ecological, evolutionary & environmental sciences

For a reference copy of the document with all sections, see nature.com/documents/nr-reporting-summary-flat.pdf

Life sciences study design

All studies must disclose on these points even when the disclosure is negative.

Sample size	NO sample size calculation was performed.
Data exclusions	No data were excluded.
Replication	Three independent replicates were performed for all studies.
Randomization	The xenograft fish were randomly allocate into groups before the treatment.
Blinding	Not relative to this study.

Reporting for specific materials, systems and methods

We require information from authors about some types of materials, experimental systems and methods used in many studies. Here, indicate whether each material, system or method listed is relevant to your study. If you are not sure if a list item applies to your research, read the appropriate section before selecting a response.

Materials & experimental systems

n/a	Involved in the study	
☐	☒	Antibodies
☐	☒	Eukaryotic cell lines
☒	☐	Palaeontology and archaeology
☐	☒	Animals and other organisms
☒	☐	Human research participants
☒	☐	Clinical data
☒	☐	Dual use research of concern

Methods

n/a	Involved in the study	
☒	☐	ChIP-seq
☐	☒	Flow cytometry
☒	☐	MRI-based neuroimaging

Antibodies

Antibodies used	Primary antibodies used include anti-DLST (H00001743, Abnova) and anti-ACTIN (sc-47778, Santa Cruz Biotechnology). Secondary antibodies include anti-mouse (31430, Thermo Scientific) or anti-rabbit antibodies (65-6120, Thermo Scientific).
Validation	Validations of antibodies were done by the indicated manufacturer and supported by the publications listed in the manufacturer's websites.

Eukaryotic cell lines

Policy information about cell lines

Cell line source(s)	All cell lines were obtained from the ATCC (www.atcc.org).
Authentication	All of the cell lines were authenticated.
Mycoplasma contamination	not contaminated.
Commonly misidentified lines (See ICLAC register)	not applicable.

Animals and other organisms

Policy information about studies involving animals; ARRIVE guidelines recommended for reporting animal research

Laboratory animals	Adult AB zebrafish, both male and female were used in the study for the xenograft.
Wild animals	Wild animals are not involved in the study.
Field-collected samples	The study did not involve samples collected from the field.
Ethics oversight	Zebrafish facility at Boston University School of Medicine, following IACUC-approved protocols.

Note that full information on the approval of the study protocol must also be provided in the manuscript.

Flow Cytometry

Plots

Confirm that:

- [ ] The axis labels state the marker and fluorochrome used (e.g. CD4-FITC).
- [ ] The axis scales are clearly visible. Include numbers along axes only for bottom left plot of group (a 'group' is an analysis of identical markers).
- [ ] All plots are contour plots with outliers or pseudocolor plots.
- [x] A numerical value for number of cells or percentage (with statistics) is provided.

Methodology

Sample preparation	Two million cells were fixed with 95% precooled ethanol, incubated overnight at -20 °C, washed with Dulbecco's Phosphate Buffered Saline (DPBS, SH30028LS, Corning), and stained with propidium iodide/RNase staining buffer (BDB550825, BD Biosciences) containing 0.1% sodium citrate for 45 minutes (min) on ice in the dark.
Instrument	Stained cells were analyzed by flow cytometry using BD LSRII SORP (BD Biosciences).
Software	ModFit LT software (Verify Software House) was used to generate DNA histograms. Each experiment was performed in triplicates.
Cell population abundance	Over 80% of cell population was used to analyze the data.
Gating strategy	To avoid the dead and aggregated cells, we gated the live cells and single cells.

- [ ] Tick this box to confirm that a figure exemplifying the gating strategy is provided in the Supplementary Information.
